# Coexisting Phosphate Controls Arsenate Speciation and Partitioning during Fe(II)-Catalyzed Ferrihydrite Transformation

**DOI:** 10.1021/acsearthspacechem.5c00061

**Published:** 2025-06-10

**Authors:** Jeffrey Paulo H. Perez, A. Li Han Chan, J. Frederick W. Mosselmans, Liane G. Benning

**Affiliations:** † GFZ Helmholtz Center for Geosciences, Telegrafenberg, 14473 Potsdam, Germany; ‡ Department of Microbiology, 196202 University of Massachusetts Amherst, Amherst, Massachusetts 01003, United States; § Diamond Light Source, Limited, Harwell Science and Innovation Campus, Didcot, Oxfordshire OX11 0DE, United Kingdom; ∥ Department of Earth Sciences, Freie Universität Berlin, 12249 Berlin, Germany

**Keywords:** adsorption, EXAFS, FTIR, green rust, magnetite, structural incorporation, vivianite

## Abstract

Arsenic immobilization in soils and sediments is primarily controlled by its sorption onto or incorporation into reactive soil minerals, such as iron (oxyhydr)­oxides. However, coexisting ions (e.g., dissolved bicarbonate, phosphate, silica, and organic matter) can negatively impact the interaction of the toxic arsenate species with iron (oxy)­hydroxides. Of special note is inorganic phosphate, which is a strong competitor for sorption sites due to its analogous chemical and structural nature to inorganic arsenate. Much of our understanding of this competing nature between phosphate and arsenate focuses on the impact on mineral sorption capacities and kinetics. However, we know very little about how coexisting phosphate will alter the stability and transformation pathways of arsenate-bearing Fe (oxyhydr)­oxides. In particular, the long-term fate and behavior regarding arsenate immobilization are unknown under anoxic conditions. Here, we document, through mineral transformation reactions, the immobilization of both phosphate (P) and arsenate [As­(V)] in secondary mineral products and characterize their changing compositions during the transformations. We did this while controlling the initial P/As­(V) ratios. Our results document that, in the absence or at low P/As­(V) ratios, the initial ferrihydrite rapidly transforms to green rust sulfate (GR_SO_4__), which further transforms into magnetite after 180 days. Meanwhile, high P/As­(V) ratios resulted in a mixture of GR_SO_4__ and vivianite, with magnetite as a minor fraction. Invariably, the speciation and partitioning of As­(V) were also affected by the P/As­(V) ratio. A higher P/As­(V) ratio also led to a faster partial reduction of mineral-bound As­(V) to As­(III). The most important finding is that the initial ferrihydrite-bound As­(V) became structurally incorporated into magnetite [low P/As­(V) ratio] or vivianite [high P/As­(V) ratio] and was thus immobilized and not labile. Overall, our results highlight the influence of coexisting phosphate in controlling the toxicity and mobility in anoxic, Fe^2+^-rich subsurface settings, such as contaminated aquifers.

## Graphical abstract

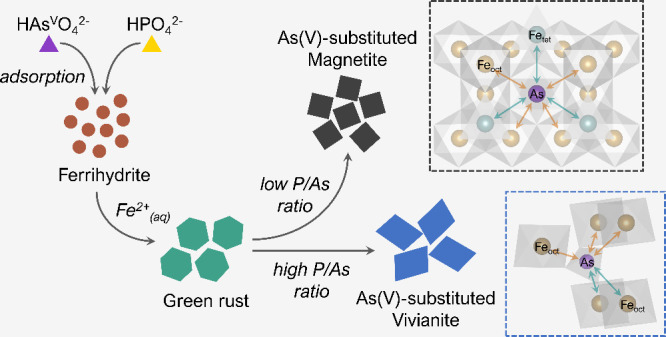

## 1 Introduction

Groundwater contamination by geogenic arsenic (As) remains a global environmental and public health concern, affecting up to 220 million people worldwide, especially in South and Southeast Asia.^1–3^ Arsenic toxicity and mobility in contaminated, reduced aquifers are controlled by the interaction of arsenic with reactive mineral phases, particularly iron (Fe)-bearing minerals.^4,5^ However, an initial immobilization of As by such Fe-bearing minerals can be again destabilized by other coexisting ions in groundwaters, leading to a *de novo* release of As into the environment.^6–8^ In particular, inorganic aqueous phosphate (PO_4_^3–^), essential to living organisms, is known to strongly inhibit As sorption onto Fe-bearing minerals.^9^ This is true particularly for inorganic arsenate [As­(V)] and arises from the chemical and structural similarity (e.g., p*K*_a_, molecular geometry) of aqueous arsenate and phosphate ions at typical circum-neutral pH conditions of groundwaters. Although the processes that affect the competitive sorption between arsenate and phosphate onto Fe­(III)-bearing minerals have been extensively studied,^9–12^ the long-term fate of the toxic arsenate associated with arsenate- and/or phosphate-bearing Fe phases in reduced soils and aquifers is still unknown.

One of the most reactive and most studied Fe­(III)-bearing mineral phases in natural and engineered environments (e.g., soils or aquifers) is ferrihydrite (FHY).^13^ FHY is a highly reactive, nanoparticulate, metastable Fe­(III) phase that readily transforms in oxic and anoxic settings to more crystalline Fe­(III) or mixed Fe­(II)/(III) phases.^13,14^ Its reactivity and persistence in the environment strongly depend on its interaction with prevalent nutrients (e.g., PO_4_^3–^), contaminants (e.g., As), or trace elements.^13,15^ For example, under reducing and circum-neutral pH conditions, FHY rapidly transforms to various, more thermodynamically stable, Fe phases due to its reaction with aqueous Fe^2+^.^16–19^ When aqueous Fe^2+^ is in excess, FHY can transform to mixed Fe­(II)/(III)-bearing phases such as green rust, magnetite, or in the presence of phosphate to the ferrous phosphate, vivianite.^20–23^

Several studies have documented how, under anoxic conditions, FHY-bound P or As­(V) influences the Fe^2+^-induced transformation of FHY (Table S1). These studies have shown that the presence of As­(V) can delay the conversion of FHY to green rust (GR) and that this inhibitory effect is stronger with increasing As/Fe ratios.^24,25^ Moreover, once GR is formed, As­(V) is known to stabilize GR, delaying its further transformation to magnetite (MGT).^24–26^ Similarly, phosphate also delays FHY transformation to GR;^20,27^ but unlike As­(V), high phosphate concentrations seem to also preferentially lead to the conversion of GR to the ferrous phosphate mineral vivianite (VIV) instead of magnetite.^23,28^ In many As-contaminated, reduced aquifers and soils,^29–33^ high phosphate levels are well-documented. However, the effect of coexisting As­(V) and phosphate on the FHY transformation pathway and the ultimate fate and mobility of mineral-bound As­(V) and/or phosphate are still poorly understood.

Here, we followed the anoxic Fe^2+^-induced transformation of FHY with adsorbed phosphate (hereafter referred to as P instead of PO_4_^3–^) and arsenate [As­(V)] at P/As ratios of 1 to 20 for up to 180 days (6 months) at pH 8. Our key objectives were to (i) reveal the impact of coexisting P and As­(V) on FHY transformation pathway, (ii) determine changes in speciation and partitioning of both P and As­(V) as FHY transforms to more thermodynamically stable secondary Fe mineral phases, and (iii) identify critical mechanisms controlling P and As­(V) immobilization in these secondary phases. To address these objectives, we combined powder X-ray diffraction, Fourier transform infrared spectroscopy, and As K-edge X-ray absorption spectroscopy to probe changes in solid-phase composition and possible changes in the oxidation state of As and the local bonding environment of P and As. We correlated the solid-state information through analyses of the changes in the aqueous chemistry of the supernatant solutions. Combining these data sets allowed us to gain a mechanistic understanding of the impact of coexisting P and As­(V) on Fe mineral transformation pathways under reducing conditions. Furthermore, our data also allows us to infer the long-term fate and stability of P and As immobilized onto Fe phases and discuss potential implications for mineral-based groundwater remediation strategies.

## 2 Materials and Methods

All glass- and plasticwares were cleaned in 1 M HCl for 24 h, followed by thorough rinsing with ultrapure water (resistivity of ∼18.2 MΩ cm). Stock solutions were prepared inside an anoxic, vinyl-walled glovebox (97% N_2_, 3% H_2_, and <1 ppm of O_2_, Coy Laboratory Products, Inc.) using deoxygenated ultrapure water obtained by purging it with argon at 90 °C for at least 4 h. Due to partial oxidation of the FeSO_4_·7H_2_O salt, Fe­(III) impurities were removed from the Fe­(II) stock solution by adding a small amount of 1 M NaOH, resulting in the precipitation of a dark green precipitate.^34^ The suspension was filtered through a 0.2 μm polycarbonate filter to obtain a clear, pale blue Fe­(II) solution. The pH of the pure Fe­(II) stock solution was adjusted to pH ∼3 using concentrated HCl. Finally, Fe­(II) concentration in the stock solution (0.58 M) was determined by inductively coupled plasma optical emission spectrometry (ICP–OES Varian 720; see Text S1).

### 2.1 Synthesis of Two-Line Ferrihydrite

Two-line ferrihydrite (FHY) was synthesized using the co-precipitation method.^13^ In an acid-cleaned perfluoroalkyl jar, 1 M NaOH was slowly added to 150 mL of 0.2 M Fe_2_(SO_4_)_3_·5H_2_O until pH ∼7 was achieved. The precipitated FHY was immediately washed by repeated cycles of centrifugation (∼10052*g* for 10 min) and resuspension in ultrapure water until the total dissolved solids concentration was <10 mg L^–1^ (Hannah Instruments). The washed FHY suspension was degassed using O_2_-free argon for at least 4 h and immediately transferred inside the glovebox. It was allowed to equilibrate with the glovebox atmosphere under stirring for at least 16 h to remove residual O_2_. FHY nanoparticles (∼3 nm in size)^35^ were not air- or freeze-dried to prevent changes in reactivity due to nanoparticle aggregation.^36^ The [Fe­(III)] in each FHY suspension was determined by ICP–OES based on the difference between the [Fe­(III)] in the suspension and supernatant. FHY suspensions ([Fe­(III) = 84.9 mM) were prepared and always used on the day of synthesis.

### 2.2 Adsorption of As and P onto FHY and Subsequent Transformation

Inside the glovebox, aliquots from 20 mM Na_2_HAsO_4_ and 20 mM Na_2_HPO_4_ (adjusted to pH 7) stock solutions were added to a 0.1 M NaCl (background electrolyte) solution to achieve [As­(V)] of 0.1 mM and [P] of 0.1 to 2 mM. This translates to desired P/As­(V) mole ratios (hereafter referred to as P/As ratios) of 1, 5, 10 and 20, which covers the range of P/As ratios found in phosphate-rich, As-contaminated aquifers in Bangladesh.^1,9^ Then, an aliquot of the FHY suspension ([Fe­(III)_FHY_ = 4 mM) was added to the mixed As­(V) and P solutions, and the pH was adjusted to ∼8 using 1 M NaOH. The mixed suspensions were stirred at 350 rpm and left to equilibrate for 24 h to allow complete adsorption of As and P (>99.9% removal) as determined by ICP–OES. To initiate the transformation of the As/P-bearing FHY suspension, aliquots of the Fe­(II) stock solution were added to achieve a final [Fe­(II)] of 8 mM and a Fe^2+^_(aq)_/Fe­(III)_FHY_ ratio of 2. This ratio, corresponding to the stoichiometric Fe­(II)/Fe­(III) ratio of GR_SO_4__, was chosen to ensure that GR_SO_4__ formation is favored over goethite,^24^ and that the subsequent transformation of GR_SO_4__ to other Fe phases proceeds fast (within days^24^). The pH of the suspensions was adjusted and then maintained at 7.0 ± 0.1 under stirring (350 rpm) using an autotitrator (TitroLine 700, YSI, Inc.). At specific time intervals (i.e., 1, 7, 15, 30, and 180 days), 10 mL aliquots of the reaction mixtures were vacuum-filtered through a 0.2 μm polycarbonate filter. Filtered solids were dried in a desiccator, ground, and stored in crimped glass vials inside the glovebox until further use. Filtered liquid phases were transferred to acid-cleaned tubes, acidified with either concentrated HNO_3_ or HCl (TraceSELECT, AristAR, VWR) for ICP–OES and ferrozine analyses, respectively, and stored at 4 °C until analyzed.

### 2.3 Analytical Techniques and Material Characterization

#### 2.3.1 Aqueous Analyses

Elemental aqueous concentrations of As, P, and Fe were determined using ICP–OES (Text S1) as done in our previous study.^37^ Limits of detection (LOD) were 0.13 μM (10 ng g^–1^) for As and 0.58 μM (18 ng g^–1^) for P. These values translate to maximum detectable removal of 99.9% for As and 99.3 to 99.9% for P. Analytical uncertainties at a 95% confidence level for concentrations quantified were <7% relative (Table S2), verified by repeat analyses of a QC solution (*n* = 8). Dissolved Fe^2+^ concentrations were determined spectrophotometrically using the ferrozine method.^38^ In this study, dissolved [Fe^2+^] measured by ferrozine analyses was equivalent to the dissolved [Fe_tot_] measured by ICP–OES.

#### 2.3.2 Powder X-ray Diffraction (XRD) and Phase Quantification

To maintain strict anoxic conditions, mineral powder samples for XRD measurements were loaded into glass capillaries (⌀ = 0.5 mm, Hilgenberg glass capillary no. 50) and sealed with Cristaseal wax (Hawksley & Sons, Ltd.) inside the glovebox. XRD patterns were recorded using a STOE STADI P X-ray diffractometer operated in Debye–Scherrer geometry using Ag Kα_1_ radiation (λ = 0.56087 Å) from a primary beam Ge(111) monochromator and equipped with two DECTRIS MYTHEN2 R 1K position-sensitive detectors. Scattered X-rays were collected over the 2θ range from 0 to ∼74°, and sample capillaries were constantly spun during data collection for improved particle statistics. The relative composition of the Fe phases was quantified by the reverse Monte Carlo (RMC) refinement method^39^ using an in-house code written in Igor Pro v.8.04 (Wavemetrics, Inc.). By applying RMC refinement, phase composition was determined by simulating the XRD pattern of a sample from a suite of reference materials, including both poorly and highly crystalline Fe phases (i.e., FHY, magnetite, GR sulfate, vivianite; Figure S1; see Text S2 for synthesis methods). Detailed reviews of the application of RMC refinement for amorphous and polycrystalline materials can be found elsewhere.^40–42^

#### 2.3.3 Fourier Transform Infrared Spectroscopy (FTIR)

FTIR spectra of the powdered samples were acquired using a Nicolet iS5 FTIR spectrometer (Thermo Fisher Scientific) equipped with a diamond attenuated total reflection (ATR) accessory (iD7 ATR). Spectra were collected over the 4000–400 cm^–1^ range with a resolution of 4 cm^–1^ by co-adding 64 individual scans. Baseline correction and peak fitting were done in the OMNIC software (Thermo Fisher Scientific). The local bonding environment of phosphate was evaluated using characteristic vibrational bands between 1200 and 880 cm^–1^, while that of arsenate was determined using bands between 880 and 680 cm^–1^. Peak assignment for deconvoluted band components was done using literature data for pure and As­(V)-substituted vivianite,^43^ and adsorbed phosphate^44–47^ and arsenate^48,49^ onto Fe (oxyhydr)­oxides, which we cross-checked with our own set of reference standards (Text S3).

#### 2.3.4 Arsenic K-edge X-ray Absorption Spectroscopy (XAS)

As K-edge XAS data were collected at beamline I20 scanning of Diamond Light Source (U.K.).^50^ Spectra were recorded at liquid nitrogen (LN_2_) temperatures (∼77 K) in transmission and fluorescence modes to a reciprocal space value of ∼14.8 Å^–1^. Fluorescence data was collected using a 64-element Ge solid-state detector equipped with Xspress4. During data collection, changes in line shape and peak position indicative of beam-induced redox reactions were examined, and no beam damage was observed. All spectra were aligned and averaged, and the background was subtracted using the SIXPack software.^51^ Shell-by-shell fits were performed were performed on the *k* ^3^-weighted extended X-ray absorption fine structure (EXAFS) spectra (*k* range = 2–12.5 Å^–1^) from 1 to 4 Å in *R* + Δ*R* space using SIXPack software^51^ based on algorithms derived from IFEFFIT.^52^ Further information on pellet sample preparation, protocols to prevent oxidation during sample transport, XAS beamline details, and data fitting procedures are described in detail in Text S4 of the Supporting Information.

#### 2.3.5 Thermodynamic and Mineral Saturation Calculations

Geochemical calculations were done in the Geochemist’s Workbench (GWB) software^53^ using the MINTEQ database. Solubility constants (p*K*_sp_) of GR phases,^54^ vivianite^55^ [Fe^II^_3_(PO_4_)_2_·8H_2_O], and parasymplesite^56^ [Fe^II^_3_(AsO_4_)_2_·8H_2_O] were manually added to the MINTEQ database based on reported data in the literature. Thermodynamically stable Fe phases (e.g., hematite, magnetite, goethite, and lepidocrocite) were suppressed successively for calculations involving thermodynamically metastable Fe phases. The reaction of HS^–^ and SO_4_^2–^ was also decoupled to model ferruginous (i.e., anoxic and non-sulfidic) conditions. The Gibbs free energies of the coupled redox transformations (Δ*G*°_rxn_) were calculated based on the Gibbs free energies of formation (Δ*G*°_f_) of corresponding Fe minerals and aqueous species (Table S3).

## 3 Results

### 3.1 Changes in Solution Chemistry during Ferrihydrite Transformation

Irrespective of P/As ratios tested, the initial aqueous [Fe^2+^] (*C*_0_ = 8 mM) decreased quickly and exhibited similar trends until the end of the transformation experiments (Figure 1A). However, the residual [Fe^2+^] after 1 day of aging was lowest (∼0.5 mM Fe^2+^) at the highest P/As ratio of 20. Nevertheless, regardless of P/As ratios, aqueous [Fe^2+^] remained relatively stable throughout the first 30 days, but slightly increased at the end of the 180-day reaction, releasing ∼5% of the solid-phase Fe^2+^ into the solution.

**Figure 1:**
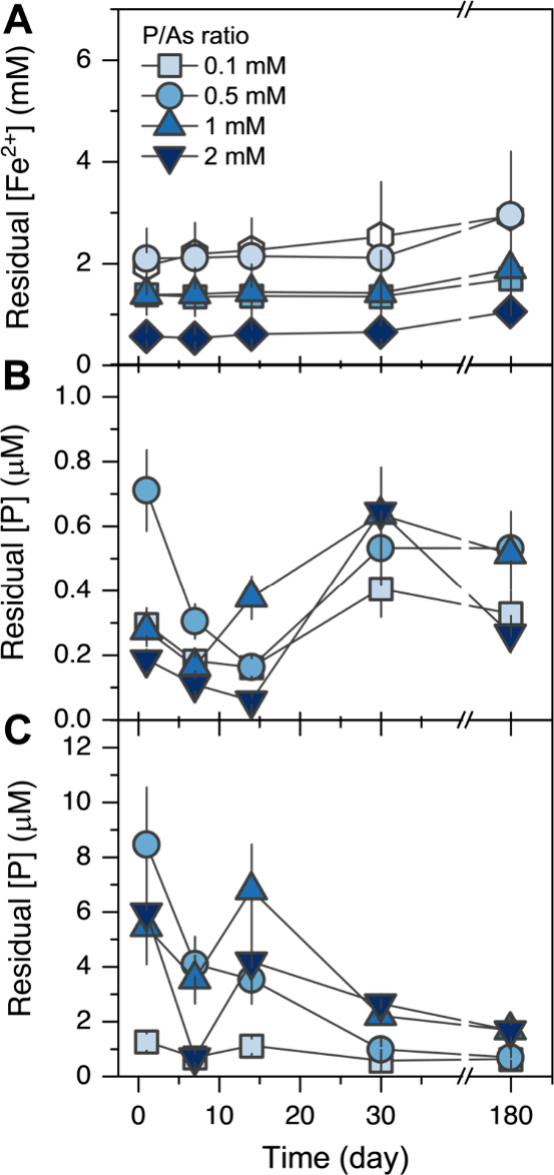
Residual aqueous (A) [Fe^2+^], (B) [As], and (C) [P] at different elapsed times during the Fe^2+^-induced transformation of FHY under anoxic conditions with varying P/As ratios. Error bars denote twice the deviation from reference values, based on replicate measurements of quality control solutions analyzed with the samples (Table S2). Note that [Fe^2+^] is reported in mM, while [As] and [P] are reported in μM.

The aqueous [As] in all the experiments remained below 0.8 μM throughout the 180-day experiment (Figure 1B). This translates to a >99.3% As removal from the initial concentration (*C*_0_ = 100 μM; Table S4). After a steady decrease within the first 14 days of transformation at all P/As ratios (except for the P/As ratio of 10), the aqueous [As] increased again to slightly higher levels (still below 0.8 mM) after 30 days, indicating the re-release of previously sorbed As. Nevertheless, throughout the 180 days of reaction, As removal efficiencies ranged from 99.5 to 99.7% for all tested P/As ratios. Interestingly, the lowest aqueous [As] was consistently observed at the highest P/As ratio of 20, while the highest aqueous [As] was determined at a P/As ratio of 5.

Finally, aqueous [P] remained consistently <9 μM (Figure 1C), equivalent to >99.3% removal in all tested P/As ratios (Table S4). The aqueous behavior of P almost mirrored the behavior of aqueous As; after almost complete adsorption, residual P concentrations further decreased in the first 7 days of the reaction, followed by P re-release before finally decreasing again until the end of the transformation reaction. However, the observed P re-release occurred at 14 days, earlier than As re-release at 30 days. In addition, it is worth noting that most As and P remained always associated with the solid mineral phases, as the removal efficiency for both anions was >99% of the initial concentrations of 100 μM As, and 100 μM to 2 mM P, respectively.

### 3.2 Solid-Phase Transformation of As­(V)/P-Bearing Ferrihydrite

In the P- and As-free experiments (control), the brown ferrihydrite (FHY) suspension changed color to dark blue–green within seconds after aqueous Fe^2+^ addition. After 1 day of aging, the XRD pattern (Figure 2A) of the separated solids contained sharp Bragg reflections associated with green rust sulfate (GR_SO_4__, 23%) and magnetite (MGT, 77%) instead of two broad reflections characteristic of the FHY precursor (Figure S1). The relative proportions of MGT and GR_SO_4__ in the aged precipitates remained relatively stable throughout the first 30 days of transformation. However, after 180 days, MGT was the only Fe phase detected in the solids, coinciding with the observed release of some of the [Fe^2+^] back into the supernatant (Figure 1A).

**Figure 2:**
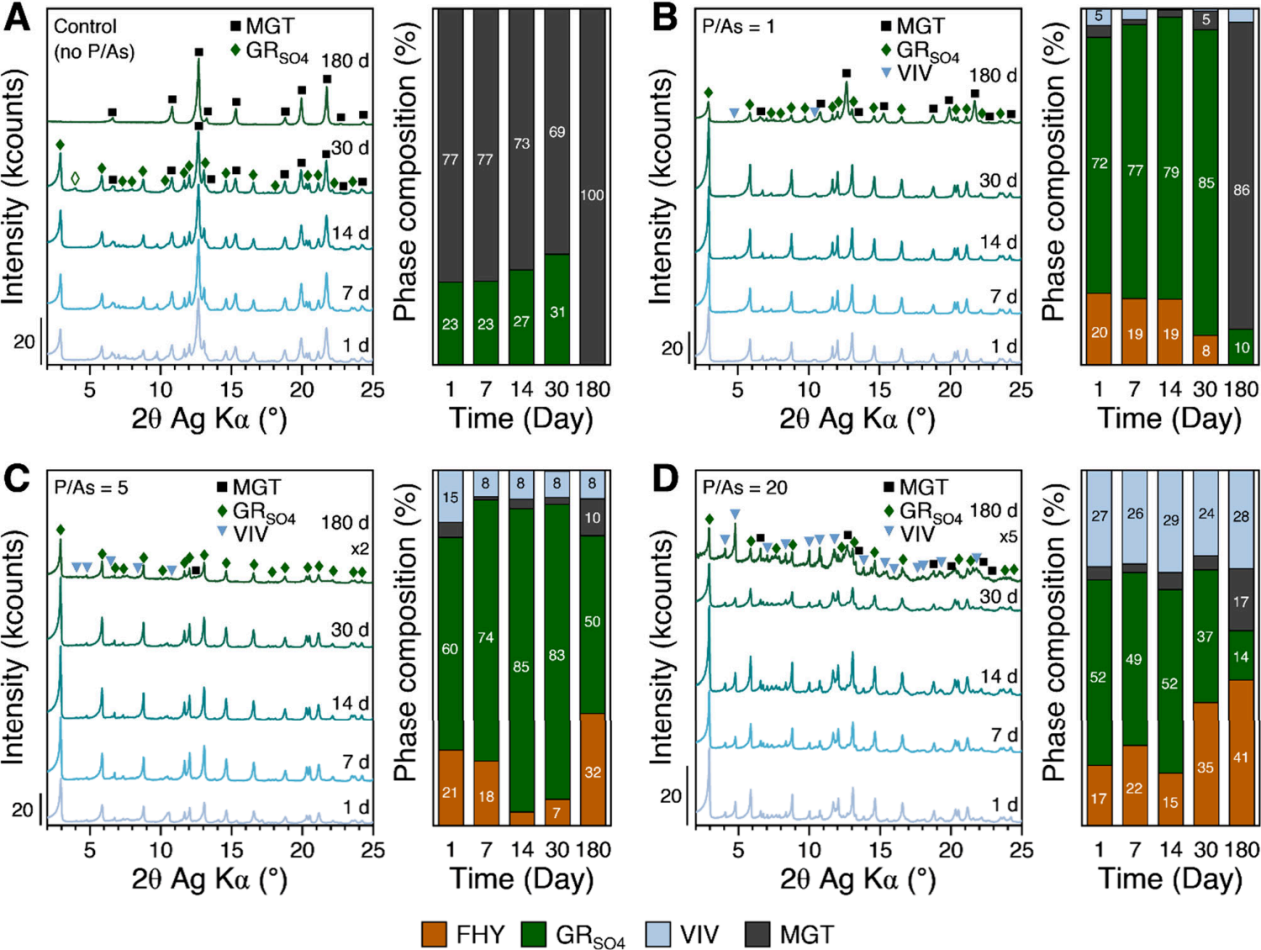
Powder XRD patterns and relative phase composition of solids collected at different elapsed times during the Fe^2+^-induced transformation of FHY under anoxic conditions with varying P/As ratios: (A) control (P- and As-free), (B) P/As = 1 ([P]_initial_ = 100 μM), (C) P/As = 5 ([P]_initial_ = 0.5 mM), and (D) P/As = 20 ([P]_initial_ = 2 mM). Bragg reflections are labeled to indicate crystalline Fe mineral phases: magnetite (MGT, black filled squares), GR_SO_4__ (green filled diamonds), and vivianite (VIV, light blue inverted triangles). Phases present below 5% relative proportion were not labeled. The minor amount of GR chloride (green hollow diamond in panel A) detected in the 30-day solids in the control is potentially a drying artifact.

Compared to the P- and As-free system, the adsorbed As and P resulted in changes in the mineral phase compositions throughout the aging experiments. At a P/As ratio of 1, the 1-day aged precipitates (Figure 2B) primarily contained GR_SO_4__ (∼72%) and the starting As–P bearing FHY material (∼20%). The presence of the initial FHY indicates that coexisting As and P delayed FHY transformation compared to the pure system. Interestingly, the solids also contained minor amounts of vivianite (VIV, ∼5%), which was not present in the P/As-free system, and some MGT (<5%). As the precipitates aged for 30 days, the proportion of GR_SO_4__ steadily increased at the expense of the initial FHY, reaching ∼85%. At this ratio, MGT (∼86%) was the dominant Fe phase at the end of the 180-day transformation experiment, yet the solids still contained small proportions of GR (∼10%) and VIV (<5%).

As the initial co-added [P] (0.5 mM) was increased 5-fold compared to [As], the amount of GR_SO_4__ formed after 1 day of aging decreased to 60% (Figure 2C), which is slightly lower than when As and P were co-added at equimolar amounts (P/As = 1). However, the proportion of VIV was higher (∼15%) compared to the reaction with P/As = 1 ratio, but the amount of remaining FHY was similar. At 14 days of aging, the amount of remnant FHY decreased to <5%, while the proportion of GR_SO_4__ increased to ∼85%. However, the amount of FHY increased again after 30 days, eventually reaching ∼32% at the end of 180 days. This sudden increase in FHY coincided with a decrease in GR_SO_4__ to ∼50% after 180 days. In addition to GR_SO_4__ and FHY, the final solids also contained small proportions of MGT (∼10%) and VIV (∼8%).

Finally, at higher P/As ratios of 10 and 20, changes in solid-phase compositions follow similar trends (Figure 2D and Figure S2). At a P/As ratio of 20, 1 day of aging yielded a mixture of GR_SO_4__ (∼52%), VIV (∼27%), unreacted FHY (∼17%) and minor amounts of MGT (<5%). Similar to the P/As ratio of 5, relative composition of these Fe phases remained the same until 14 days but changed again afterward. In particular, the proportion of FHY in the resulting solids increased again at the expense of GR_SO_4__. After 180 days, the solids contained FHY (∼41%), VIV (∼28%), MGT (∼17%), and GR (∼14%).

### 3.3 Phosphate Local Bonding Environment

To determine the local bonding environment of phosphate in all solid samples collected after 7, 30, and 180 days at P/As ratios of 1 and 20, we deconvoluted the Fourier transform infrared (FTIR) spectra, particularly the bands of interest between 1200 and 880 cm^–1^ (more details in Text S3).

At a P/As ratio of 1, the FTIR spectrum of the solids collected after 7 days of reaction could be best fitted with 8 component bands (Figure 3A). The 4 component bands at 1131, 1102, 1082, and 1039 cm^–1^ (green bands) match the sulfate stretching bands [ν­(SO_4_)] from the SO_4_^2–^ interlayer anions of GR_SO_4__.^57,58^ The remaining component bands at 1061, 1014, and 987 cm^–1^ could be assigned to the phosphate stretching bands [ν­(PO_4_)], corresponding to adsorbed phosphate species.^44,47^ The position of the ν­(PO_4_) bands agrees with the reported values for phosphate adsorbed onto iron minerals in a bidentate binuclear (^2^ *C*) geometry (red bands). The additional band at 896 cm^–1^ corresponds to the lattice Fe–OH vibration of GR_SO_4__.^57,58^ After 30 days of reaction, the number and positions of the fitted component bands did not change (Figure 3B), but the integrated band areas increased by ∼1.5-fold for all components (Table S6). However, in the samples collected at the end of the 180-day reaction period, the shape of the FTIR spectrum (Figure 3C) changed noticeably, which is also mirrored in the differences in the fitted component bands. A new band at 1156 cm^–1^, assigned to the ν­(P=O) stretching band of phosphate,^44^ may reflect changes in the local bonding environment of P associated with magnetite. Specifically, P may substitute for tetrahedral Fe^3+^ sites in the magnetite lattice,^59^ distorting the PO_4_ geometry and potentially leading to corresponding shifts in vibrational modes. Moreover, instead of the 4 component bands observed after 7 and 30 days, only two bands (1130 and 1105 cm^–1^) could be attributed to ν­(SO_4_) of GR_SO_4__, which is not surprising since the proportion of GR_SO_4__ had decreased to ∼10% (Figure 2B). The bands at 1046, 985, and 925 cm^–1^ (blue bands) match the reported positions of the ν­(PO_4_) of phosphate in the crystal structure of vivianite.^43,60,61^ The remaining component bands at 1079, 1015, and 959 cm^–1^ were again attributed to the adsorbed phosphate species in ^2^ *C* geometry.

**Figure 3:**
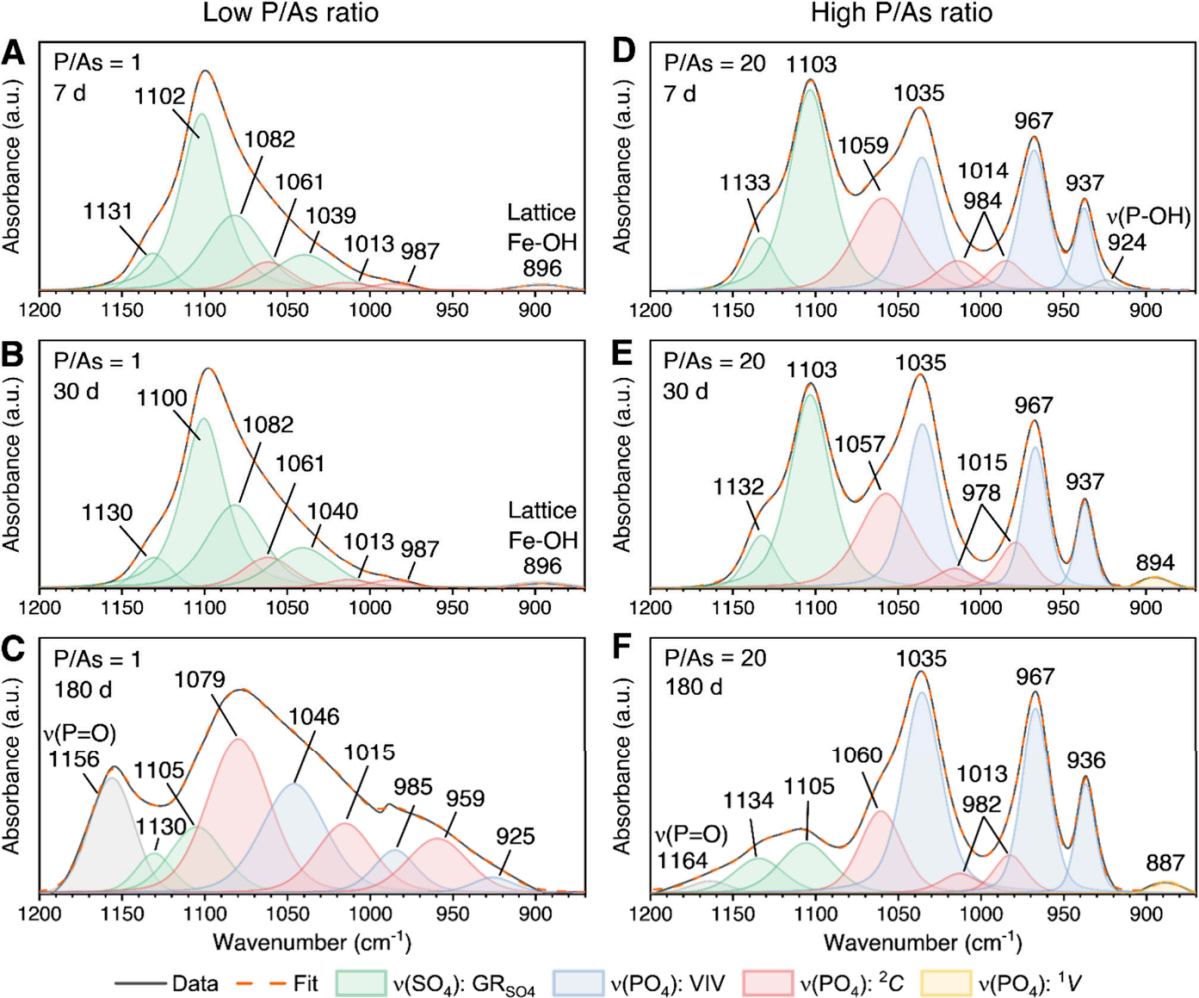
Deconvoluted FTIR spectra [ν­(PO_4_) region: 1200–880 cm^–1^] of solids collected at different elapsed times at P/As ratios of (A–C) 1 and (D–F) 20. Fitted component bands consist of phosphate stretching bands [ν­(PO_4_)] assigned to (i) bidentate binuclear complexes (^2^ *C*; red), (ii) monodentate mononuclear complexes (^1^ *V*; yellow), and (iii) vivianite (VIV; blue). The sulfate stretching bands [ν­(SO_4_)] from the GR_SO_4__ are shown in green. The total fit (orange dashed lines) is superimposed on the experimental data (black solid line), and the fitting results and further statistics are given in Tables S5 and S6.

In the case of the P/As ratio of 20, deconvolution of the FTIR spectrum of the 7 day reacted solids (Figure 3D) revealed component bands consisting of ν­(SO_4_) of GR_SO_4__ (1133 and 1103 cm^–1^) and ν­(PO_4_) from vivianite (1035, 967, and 937 cm^–1^) and adsorbed phosphate (^2^C geometry; 1059, 1014, and 984 cm^–1^). An extra band component at 924 cm^–1^ was assigned to the ν­(P–OH) stretching vibration of HPO_4_^–^ species.^62^ This band disappeared with aging, indicating a shift in P speciation, likely due to transformation of mineral-bound HPO_4_^–^ into more stable forms, such as structural incorporation into newly formed vivianite. By 30 days, the fitted band components were similar to the 7-day reacted sample with ∼10% increase in band areas for ν­(SO_4_) of GR_SO_4__ and ν­(PO_4_) of adsorbed phosphate (Figure 3E and Table S7). A new band at 896 cm^–1^ also appeared, representing adsorbed phosphate in a monodentate mononuclear (^1^ *V*) geometry (yellow band). However, after 180 days, the band area of ν­(SO_4_) of GR_SO_4__ decreased significantly (∼69%) compared to the 30-day reacted solids, which agrees with the XRD data that documented smaller proportions of GR_SO_4__ in the solid phases (Figure 2D).

### 3.4 Arsenic Oxidation State and Its Local Bonding Environment

Arsenic K-edge X-ray absorption near edge spectra (XANES; Figure 4) acquired from the solids derived from experiments at P/As ratios of 1 and 20 at different reaction stages allowed us to probe any changes in the oxidation state of mineral-bound As during the transformation. For P/As ratio of 1 (Figure 4A), only after 180 days could the reduction of the initial As­(V) to As­(III) (∼15%) be observed. In contrast, at P/As ratio of 20 (Figure 4B), ∼7% of the initial As­(V) was already reduced to As­(III) within 30 days (Table S8), which is six times faster than the reduction observed at the lowest P/As ratio tested. After 180 days of reaction, the degree of reduction of mineral-bound As increased to reach ∼26% (Table S8), which is almost twice compared to those observed at P/As ratio of 1 after 180 days of reaction. In comparison, the oxidation state of As­(III) and As­(V) (*C*_0_ = 100 μM) co-precipitated with synthetic ferrihydrite (FHY), green rust sulfate (GR_SO_4__), and magnetite (MGT) did not change.

**Figure 4:**
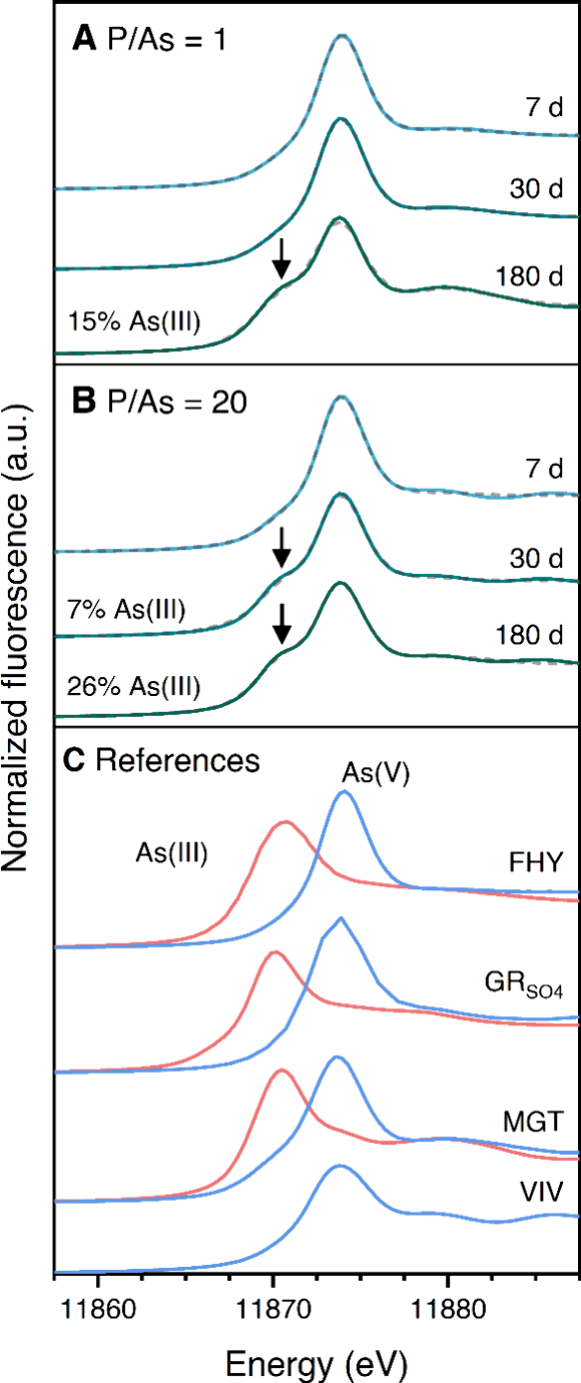
As K-edge XANES spectra of solids collected at different elapsed times at P/As ratios of (A) 1 and (B) 20. Arrows denote the reduction of the initial As­(V). Dashed lines indicate linear combinations of XANES reference spectra from As­(III) and As­(V) adsorbed on FHY. (C) Reference spectra for vivianite (VIV) and As­(III) (red patterns) and As­(V) (blue patterns) adsorbed onto FHY, GR_SO_4__, and MGT are shown for comparison.^43,58,63^

Changes in the local bonding environment of mineral-bound As at both lowest and highest P/As ratios of 1 and 20 were monitored through changes in the As K-edge extended X-ray absorption fine structure (EXAFS; Figure 5A) analysis. At a P/As ratio of 1, the *k* ^3^-weighted EXAFS spectrum of the solids collected after 7 days exhibited smooth oscillations similar to features found in As­(V) co-precipitated with GR_SO_4__ (green spectra labeled GR_SO_4__ in the bottom references panel in Figure 5A). The same smooth features were observed after 30 days of reaction. However, at the end of the experiment (180 days), the EXAFS spectrum exhibited three beat features at 6.0, 7.9, and 8.3 Å^–1^, and two prominent shoulders at 6.8 and 10.2 Å^–1^ in the second and third oscillations, respectively. These distinctive features, marked by inverted triangles in Figure 5A, match those observed in As­(V) co-precipitated with magnetite (dark gray spectra labeled MGT in the bottom references panel in Figure 5A).

**Figure 5:**
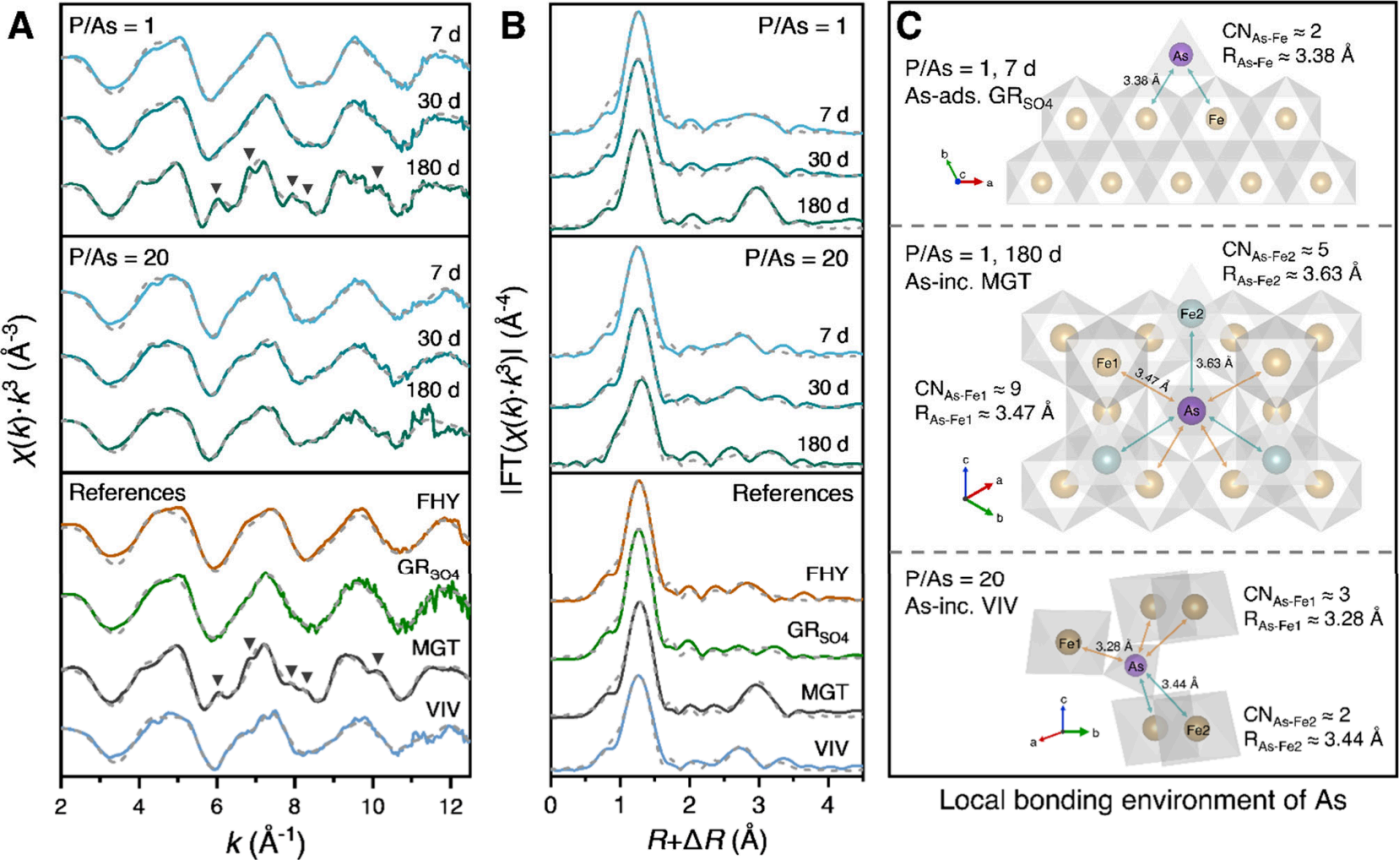
(A) As K-edge *k* ^3^-weighted extended EXAFS spectra of the solids collected at different elapsed times at P/As ratios of 1 (top panels), P/As ratios of 20 (middle panels), and the reference phases (bottom panels) that contain As­(V) co-precipitated with FHY, GR_SO_4__, MGT, and VIV (see Text S2 for details). (B) Fourier-transformed EXAFS spectra. Shell-by-shell fitting outputs (gray dashed lines) are superimposed on experimental data (colored solid line); fitting results are given in Table S9. (C) Structural models showing the different immobilization mechanisms of arsenic into secondary Fe­(II)-bearing minerals: (top) As adsorption onto green rust sulfate (GR_SO_4__) crystal edges as binuclear bidentate, inner-sphere surface complexes (^2^ *C*); (middle) partial As incorporation into the magnetite (MGT) structure by replacement of tetrahedral Fe­(III); and (bottom) partial As incorporation into vivianite (VIV) by replacement of tetrahedral P.

Shell-by-shell fits of the Fourier-transformed EXAFS spectra (Figure 5B and Table S9) allowed us to quantify the local bonding environment of mineral-bound As. In the solids collected after 7, 30, and 180 days at a P/As ratio of 1, the shell-by-shell fits of the first neighbor contribution yielded a coordination number (*CN*) of 4.3–4.7 at an interatomic distance (*R*) of 1.69–1.71 Å, fitting the As^V^O_4_ tetrahedra. Fits of the second neighbor contribution required a single As–Fe path (i.e., atomic correlations) for the 7- and 30-day solids with *R*_As–Fe_ values of 3.38–3.40 Å. These values are close to the As–Fe atomic correlations found in As­(V) co-precipitated with FHY and GR_SO_4__, wherein As­(V) is adsorbed as a bidentate, binuclear (^2^C) inner-sphere complex^63–66^ (Figure 5C, top structure). Meanwhile, two As–Fe paths (*R*_As–Fe1_ ≈ 3.47 Å, *R*_As–Fe2_ ≈ 3.63 Å) were needed for the solids collected after 180 days, matching with the As–Fe paths determined for As­(V) co-precipitated with MGT. These *R*_As–Fe_ values match the Fe–Fe interatomic distances of tetrahedral Fe­(III) in the crystal structure of magnetite^67^ (Figure 5C, middle structure), suggesting partial As­(V) substitution for Fe­(III).^24,68,69^ Complementary to the EXAFS data, the region between 880 to 780 cm^–1^ of the FTIR spectra also provides additional local bonding information on mineral-bound As. Unfortunately, this overlaps with lattice Fe–OH vibrations of GR_SO_4__, the dominant Fe phase at a P/As ratio of 1 (Figure 2B), making band deconvolution and assignment challenging (Figure S4 and Table S10; more details in Text S3). Nonetheless, deconvolution of the FTIR spectra of the 7- and 30-day solids (Figure S5) revealed a ν­(As–OH) band at ∼753 cm^–1^, which can be assigned to adsorbed HAs^V^O_4_^2–^ in ^2^ *C* geometry^70–72^ and agrees with our As K-edge data. After 180 days, the shape of the FTIR band in this region changed dramatically, reflecting the complete transformation of GR_SO_4__ to MGT (Figure 2B). Band deconvolution (Figure S5 and Table S12) revealed three component ν­(AsO_4_) bands at ca. 826, 812, and 798 cm^–1^ that correspond to adsorbed protonated and deprotonated As­(V) bound to Fe polyhedra^73–75^ and two bands at ca. 785 and 772 cm^–1^, attributed to ν­(AsO_3_) from Fe mineral-bound As­(III),^76^ arising from the partial oxidation of As­(V) (Figure 4A).

At a P/As ratio of 20, the EXAFS spectra of all solids, irrespective of aging time, exhibited a broad shoulder in the second oscillation at ∼7 Å (Figure 5A). This resembles the feature found in As­(V) co-precipitated with FHY and vivianite (bottom reference panel in Figure 5A). Shell-by-shell fits of the first neighbor contribution of the EXAFS spectra also yielded *CN*_As–O_ and *R*_As–O_ values indicative of the As^V^O_4_ tetrahedra (Table S9). The second neighbor contribution was fitted with 2 As–Fe interatomic correlations at 3.28–3.30 and 3.43–3.47 Å, again distances similar to the *R*_As–Fe_ values in the As­(V) co-precipitated vivianite (*R*_As–Fe1_ ≈ 3.29, *R*_As–Fe2_ ≈ 3.46 Å) where As­(V) is structurally incorporated by replacing phosphate^43,77^ (Figure 5C, bottom structure). In agreement with the EXAFS data, fitted component bands of the FTIR spectra of 7- and 30-day solids (Figure S5) could all be assigned to As­(V)-incorporated vivianite (Table S10). Bands at ∼846, ∼821, and ∼746 cm^–1^ correspond to ν­(As–O) vibrations, while bands at ∼777 and ∼725 cm^–1^ are linked to ν­(As–O–Fe) vibrations (Table S11). It is important to note that these band positions are red-shifted compared to As­(V)-substituted vivianite (48 mol % As substitution; Figure S4) reference spectra due to the lower degree of As­(V) substitution in the reacted solids in this work (<4 mol % As substitution; Table S4). However, as the proportion of As­(V)-incorporated vivianite decreased at the end of 180 days, the shape of the arsenate stretching band changed (Figure S5), which is also reflected in the deconvoluted component bands (Table S11). Some of the bands assigned to As­(V)-substituted vivianite disappeared concomitant to the appearance of new bands at ∼817 and ∼767 cm^–1^, corresponding to adsorbed HAs^V^O_4_^2–^ and H_3_As^III^O_3_^0^ species, respectively.

## 4 Discussion

We investigated the effect of coexisting P and As­(V) on the Fe^2+^-induced transformation of FHY under anoxic conditions at P/As ratios varying between 1 and 20 over 180 days. Our results clearly document that the presence of P altered the pathway of the Fe^2+^-induced transformation of pure or As-bearing FHY. Without both As and P species, FHY rapidly transformed to GR_SO_4__ and further to the more thermodynamically stable magnetite (MGT) (Figure 2A). This is expected because MGT is the thermodynamically stable Fe phase in our tested systems and matches our geochemical calculations (see saturation indices in Table S14). This Fe^2+^-induced transformation pathway under anoxic conditions (FHY → GR → MGT) has already been established in many earlier studies.^22,78,79^ The presence of adsorbed equimolar P and As­(V) in the FHY system resulted in a substantial delay and an incomplete transformation of the FHY precursor even after 30 days of reaction; furthermore, the coexistence of P and As­(V) also extended the lifetime of the metastable GR_SO_4__ intermediate up to 30 days (Figure 2B). This is a consequence of the stabilizing effect of phosphate and arsenate being bound as inner-sphere surface complexes (panels A and B of Figure 3 and panels B and C of Figure 5) at the GR_SO_4__ crystal edges, which inhibits its dissolution and further transformation to the stable magnetite.^63^ Our results show that the co-addition of P to As­(V)-bearing FHY results in a compound inhibitory effect (either additive or synergistic) on both the GR_SO_4__ crystallization from FHY and its persistence and inhibition against the inevitable subsequent conversion to magnetite. However, since we did not test single As­(V) or P-bearing FHY transformations in this study, we cannot definitively say whether the observed inhibitory effect was additive or synergistic. A similar effect has previously been documented for Si co-added to As­(V)-bearing FHY^58^ wherein the GR_SO_4__ intermediate was only stable for <1 day with As­(V) only and <15 days with Si only, transforming to MGT afterward. However, coexisting As­(V) and Si allowed GR_SO_4__ to resist MGT conversion for >30 days, and potentially even up to >1 year under natural groundwater conditions with comparable As and Si concentrations.^80^

After 180 days, almost all GR_SO_4__ was converted to magnetite (Figure 2B), and the initially adsorbed As­(V) at GR_SO_4__ crystal edges^63^ became incorporated in the magnetite structure (Figure 5C). Interestingly, mineral-bound As­(V) was partially reduced, with ∼15% As­(III) detected after 180 days. An earlier study from van Genuchten^68^ also reported partial As­(V) reduction (up to ∼30%) during GR_CO3_ conversion to magnetite over 150 days. So far, identifying the electron donor in these mixed valence Fe transformations has been challenging because studies involving adsorption^63,81^ or co-precipitation (Figure 3C) of As­(V) with freshly precipitated GR or magnetite have not resulted in redox changes. Our thermodynamic calculations (Table S15 and eq 1), however, suggest that magnetite (Fe^II^Fe^III^_2_O_4_) could reduce As­(V) to As­(III), leading to its partial oxidation to maghemite (γ-Fe^III^_2_O_3_).

1 $${2\mathrm{Fe}}^{\mathrm{II}}{{\mathrm{Fe}}_{2}}^{\mathrm{III}}O_{4}+{2\mathrm{HAs}}^{V}{O_{4}}^{2-}+{4H}^{+}⇌3\gamma {{\mathrm{‐Fe}}_{2}}^{\mathrm{III}}O_{3}+{2\mathrm{As}}^{\mathrm{III}}{\left(\mathrm{OH}\right)}_{3}+H_{2}O$$

We therefore speculate that the presence of aqueous Fe^2+^ (∼2 mM; Figure 1A) enhanced the reductive capability of magnetite^82–84^ compared to a pure magnetite system. Our findings support the recent study of van Genuchten,^68^ and could indicate that structural incorporation and long reaction times are both critical requirements for As­(V) reduction in magnetite–Fe^2+^_(aq)_ suspensions, irrespective of the initial [Fe^2+^_(aq)_] (cf. Huhmann et al.,^69^ Gubler and ThomasArrigo^85^ and Perez et al.^24^). It is important to note that the low As­(V) concentration used in this study would likely have resulted in a non-stoichiometric magnetite rather than maghemite; however, further experiments are needed to determine the validity of this hypothesis and to resolve the structure of the resulting non-stoichiometric magnetite end product.

The second interesting finding in our work was the observation that with increasing co-added [P] compared to [As­(V)], vivianite started to form (Figure 2). The proportion of vivianite was, however, constrained, only reaching 28 wt %, even at the highest P/As ratio, and even though, based on thermodynamic calculations (Table S9), vivianite is more stable than GR_SO_4__. Notably, while coexisting P and As lead to the incomplete transformation of the initial FHY, an increase in FHY proportion, as observed in the late stages of the reactions (i.e., after 30 days) at P/As ratios ≥ 5, was unexpected. This increased FHY fraction matched both the partial reduction of mineral-bound As­(V) to As­(III), which reached 26% at the end of 180 days (Figure 4B), as well as a minor re-release of initially immobilized As (<1% of [As]_initial_) in the supernatant (Figure 1B). A study by Xiong et al.^28^ proposed that at high [P] [P/Fe­(II) < 30], GR transforms to vivianite via a dissolution–precipitation mechanism while also generating P-bearing FHY *de novo*. While this could explain the appearance of FHY at the expense of GR_SO_4__ in our experiments (Figure 2), the proportion of vivianite did not concomitantly increase in the later stages of our reactions. Moreover, the relative proportion of vivianite at all tested P/As ratios remained relatively stable throughout 180 days. The release of FHY-bound P, a rate-limiting step during precipitation, could explain its low proportion in the solids. However, it is highly likely that As­(V) was reduced to As­(III) by vivianite (Δ*G*°_rxn_ = −131.3 to −209.5 kJ mol^–1^; Table S15), as shown in eqs 2 and ([Disp-formula eq3]), resulting in the formation of FHY *de novo*.

2 $${{\mathrm{Fe}}_{3}}^{\mathrm{II}}{(\mathrm{PO}}_{4}{)}_{2}{\cdot 8H}_{2}O+{3\mathrm{HAs}}^{V}{O_{4}}^{2-}+{4H}^{+}⇌{3\mathrm{Fe}}^{\mathrm{III}}{\left(\mathrm{OH}\right)}_{3}+{2H}_{2}{{\mathrm{PO}}_{4}}^{-}+{3\mathrm{As}}^{\mathrm{III}}{\left(\mathrm{OH}\right)}_{3}+{2H}_{2}O$$

3 $${{\mathrm{Fe}}_{3}}^{\mathrm{II}}{(\mathrm{PO}}_{4}{)}_{2}{\cdot 8H}_{2}O+{3\mathrm{HAs}}^{V}{O_{4}}^{2-}⇌{3\mathrm{Fe}}^{\mathrm{III}}{\left(\mathrm{OH}\right)}_{3}+2{{\mathrm{HPO}}_{4}}^{2-}+{3\mathrm{As}}^{\mathrm{III}}{\left(\mathrm{OH}\right)}_{3}+{2H}_{2}O+{4H}^{+}$$

The proposed partial reduction of As­(V) by vivianite and subsequent formation of FHY would also explain the re-release of As­(III) at 30 days. Compared to As­(V), As­(III) typically has a lower sorption affinity for Fe minerals^9^ and would, therefore, take more time to get sequestered in newly formed FHY.^24^ More importantly, at a P/As ratio of 20, As­(V) remained strongly bound within vivianite, being tightly confined inside their crystal structures and thus preventing its remobilization.

## 5 Conclusion

We demonstrated through complementary solid and solution analyses that inorganic phosphate coexisting with arsenate controls two critical processes during the Fe^2+^-induced transformation of P and As-bearing FHY under anoxic conditions: (i) the presence of inorganic P hinders the mineral transformation pathway and changes resulting mineral phases and (ii) the presence of inorganic P affects both the arsenic speciation and its immobilization mechanism in stable mineral phases. We show that two potential mineral sinks for As­(V) in eutrophic and ferruginous environments: As­(V)-substituted magnetite form at low levels of coexisting phosphate (P/As < 5), whereas As­(V)-substituted vivianite persists at high levels of coexisting phosphate (P/As ≥ 5).

Our findings indicate that within the context of eutrophic environments, magnetite and vivianite are thermodynamically stable Fe­(II)-bearing phases that can act as favorable, stable sinks for removing As­(V) in ferruginous settings. In many soils, as well as in lacustrine and coastal environments, magnetite^86–89^ and vivianite^90–94^ are widely recognized as key phosphorus (P) sinks, playing a crucial role in regulating its (bio)­availability and mobility. Although As substitution has not been reported in natural magnetite and vivianite, several studies have shown that synthetic analogues can incorporate As­(V) into their crystal structures, up to 0.8–2 wt % for magnetite^24,68,81^ and as much as 24 wt % in vivianite–parasymplesite solid solutions.^43,77^

Furthermore, this study has implications for understanding mineral-based technologies for As remediation and P recovery. The results obtained at low P levels support the viability of *in situ* magnetite precipitation, either by electrocoagulation^68^ or (bio)­crystallization,^95^ as a long-term approach for As immobilization. Our findings at elevated P levels also indicate the potential for integrating As remediation with P recovery through vivianite (bio)­crystallization technology,^96,97^ particularly in eutrophic, As-contaminated natural and engineered environments (i.e., aquifers, lakes, and mining areas).

## Supplementary Material



## Notes

Details on mineral synthesis, experimental and analytical methods, XRD patterns of reference Fe phases, additional XRD and solid-phase quantification data, deconvolution of the FTIR spectra of reference phases, previous studies on As/P-bearing ferrihydrite transformation, analytical uncertainties and instrumental limits, band assignment of FTIR spectra of samples and reference phases, LCF results and statistics, mineral saturation indices, and supplementary Gibbs free energies of potential redox reactions and standard Gibbs free energy of formation (PDF)
