# Supplementary material for: Coexisting Phosphate Controls Arsenate Speciation and Partitioning during Fe(II)-Catalyzed Ferrihydrite Transformation
Source: ACS Earth Space Chem. 2025 Jun 10;9(6):1642–53. doi: 10.1021/acsearthspacechem.5c00061 (PMC12183708; doi:10.1021/acsearthspacechem.5c00061)
Supplement: Supplementary file 1 [file sp5c00061_si_001.pdf]

SUPPORTING INFORMATION

for

Coexisting phosphate controls arsenate speciation and partitioning during Fe(II)-catalyzed ferrihydrite transformation

Jeffrey Paulo H. Perez[a,*], A. Li Han Chan[a,b], J. Frederick W. Mosselmans[d], Liane G. Benning[a,e]

[a] GFZ Helmholtz Centre for Geosciences, Telegrafenberg, 14473 Potsdam, Germany

[b] Department of Microbiology, University of Massachusetts Amherst, Amherst, MA 01003, USA

[c] Diamond Light Source Ltd., Harwell Science and Innovation Campus, Didcot, Oxfordshire OX11 0DE, United Kingdom

[d] Department of Earth Sciences, Freie Universität Berlin, 12249 Berlin, Germany

* Corresponding author. E-mail address: jpperez@gfz.de (J.P.H. Perez)

This Supporting Information includes 27 pages, 15 tables, and 5 figures.

	Page
Aqueous elemental concentration analysis	S1
Synthesis of reference minerals	S2
Fourier transform infrared spectroscopic analyses of solid phases	S3
Arsenic K-edge X-ray absorption spectroscopic characterization of solids	S4
Supporting Figures	S5
Supporting Tables	S10
Supporting References	S25

Text S1. Aqueous elemental concentration analysis

The supernatants were separated from the solids in each experiment by vacuum filtering the suspensions through a 0.2-μm polycarbonate membrane filter and acidifying the resulting fluids with concentrated HNO_3 (TraceSELECT™, AristAR®, VWR). Acidified aqueous samples were stored at ~4 °C until analysis. Samples (pH ~2) were diluted gravimetrically in acid-cleaned polypropylene (PP) tubes before inductively coupled plasma optical emission spectrometry (ICP-OES) analyses. Sample dilutions were performed using 0.3 M HNO_3 (Merck Suprapur® grade) containing 1 mg g^{-1} Cs as an ionization buffer to achieve matrix matching with calibration standards prepared from a mixture of single ICP element standards (Merck Certipur®, traceable to NIST reference materials). Scandium (1 μg g^{-1}) was added as an internal standard. Dilution factors were adapted to ensure that analyte solutions were within the concentration range of the matrix-matched calibration standards (linearity criteria $R^2 > 0.9990$) and with final HNO_3 and Cs concentrations of 0.3 M and 1 mg g^{-1}, respectively.

For each analytical session, instrumental stability and drift were monitored using Ar, Cs, and Sc at emission wavelengths of 420.067, 459.311, and 335.372 nm for each sample analysis, respectively, as well as regular measurements of a quality control (QC) solution, similar to sample compositions. Instrumental statistical limits of detection (LOD = 3SD above background) or limits of quantification (LOQ = 10SD above background) were determined in each analytical session based on nine repeat analyses of 0.3 M HNO_3 (+ 1 mg g^{-1} Cs) used for sample dilution.

Aqueous total As, P, and Fe concentrations were evaluated using the emission wavelengths of 193.696, 177.434, and 261.382 nm, respectively. The determined LOD values were 10 ng g^{-1} for As, 18 ng g^{-1} for P, and 4 ng g^{-1} for Fe. Analytical uncertainties at a 95% confidence level for concentrations quantified (above LOQ) during this study are <7% relative, verified by repeat analyses of a QC solution, which was similar to the sample compositions (see Table S2).

Text S2. Synthesis of reference minerals

Pure ferrihydrite (FHY). FHY was prepared by slowly adding 1 M NaOH to 150 mL of 0.2 M $Fe_2(SO_4)_3 \cdot 5H_2O$ until pH ~7. The brown FHY suspension was centrifuged (~10,052×*g* for 10 min) and then resuspended in ultrapure water, which was repeated until the total dissolved solids were <10 mg L^{-1}.

P adsorbed on FHY. In an acid-cleaned serum bottle, 100 mg of freeze-dried FHY powder was resuspended in 50 mL of Milli-Q water by shaking at 50 rpm for 16 h using an orbital shaker. Afterward, 50 mL of 4 mM PO_4^{3-} (buffered at pH 7 using NaH_2PO_4-Na_2HPO_4) were added to the ferrihydrite suspension, translating to a final [P] of 2 mM and $[Fe]_{FHY}$ of 9.4 mM (P/Fe = 0.21). The mixture was shaken at 250 rpm for 24 h at room temperature in an orbital shaker.

As(V)-coprecipitated FHY. In a 1-L PFA reactor, we added 15.6 mg $Na_2HAsO_4 \cdot 7H_2O$ (0.05 mmol) to a 1 L of 5 mM $Fe(NO_3)_3 \cdot 9H2O$ solution (5 mmol). The pH of the mixed solution was adjusted from ~3 to ~7.5 using 1 M NaOH. The resulting suspension was washed through three cycles of centrifugation (10,595*g* for 5 min) and resuspension in Milli-Q water.The following reference phases are susceptible to oxidation in air. Therefore, experiments were performed inside a vinyl-walled glovebox (97% N_2, 3% H_2, Coy Laboratory Products, Inc.). Stock solutions were prepared using O_2-free ultrapure water.

Green rust sulfate (GR_{SO4}). GR_{SO4} was prepared by mixing 50 mL of 0.3 M $Fe(NH_4)_2(SO_4)_2 \cdot 6H_2O$ and 50 mL of 0.1 M $Fe_2(SO_4)_3$, followed by dropwise addition of 100 mL of 0.3 M NaOH.[1] The final pH of the dark blue-green suspension is ~7.2. As(V)-coprecipitated GR_{SO4} was obtained by aging a slurry of As(V)-adsorbed ferrihydrite (As/Fe = 0.025) in the presence of 12 mM $Fe^{2+}_{(aq)}$ at pH 8 and at a $Fe^{2+}_{(aq)}/Fe(III)_{FHY}$ ratio of 3.

Vivianite. Vivianite was synthesized by mixing 50 mL of a 10 mM $Fe(NH_4)_2(SO_4)_2 \cdot 6H_2O$ solution and 50 mL of a 10 mM PO_4^{3-} solution buffered at pH 7.2.[2] The final pH of the immediately formed light blue suspension was ~6.4. To produce As(V)-incorporated vivianite (~48 mol% substitution), 25 mL of 10 mM Na_2HAsO_4 solution (pH ~7) was mixed with 25 mL of a 10 mM PO_4^{3-} buffer solution, followed by the addition of 50 mL of a 10 mM $Fe(NH_4)_2(SO_4)_2 \cdot 6H_2O$ solution. The mixed light blue-green suspension had a final pH of ~6.5.

Magnetite. Magnetite was synthesized by mixing 100 mL of a 5 mM $FeCl_2 \cdot 4H_2O$ solution and 100 mL of a 10 mM $FeCl_3 \cdot 6H_2O$ solution , followed by dropwise addition of a 1 M NaOH solution until a pH between 10-11 was reached and a black precipitate formed.[3] As(V)-incorporated magnetite was obtained by aging a slurry of As(V)-adsorbed ferrihydrite (As/Fe = 0.025) in the presence of 12 mM $Fe^{2+}_{(aq)}$ at pH 8 and at a $Fe^{2+}_{(aq)}/Fe(III)_{FHY}$ ratio of 3 for 30 days.[4]

All resulting mineral suspensions were aged for 1 h under stirring (350 rpm) and then vacuum filtered through 0.2-μm polycarbonate membrane filters unless otherwise stated. The collected solids on the filters were dried in a petri dish placed inside a desiccator inside the glovebox. After ~24 h, the mineral solids were finely ground using an agate mortar and pestle and transferred and stored inside crimp-capped vials inside the glovebox to maintain anoxic conditions. The purity of the mineral reference materials was confirmed through XRD and/or FTIR spectroscopic analyses (see Figures S1, S3, and S4).

Text S3. Fourier transform infrared (FTIR) spectroscopy.

Aliquots of the dry powder samples were transferred to 2-mL glass vials inserted into 20-mL crimp-capped glass vials to prevent oxidation during transport from the glovebox to the FTIR equipment.

Sample vials were opened just before each spectrum was collected. Although the FTIR spectra were collected over the 4000–400 cm^{-1} range, the regions of interest are: (i) 1200–880 cm^{-1} covering the characteristic vibrational bands for phosphate and (ii) 880–680 cm^{-1} containing the vibrational bands for arsenate. We considered three possible mineral-bound phosphates and arsenates: (i) adsorbed species as inner-sphere surface complexes in bidentate binuclear (^2C) or monodentate mononuclear (^1V) geometry; and (ii) incorporated in the crystal structure of iron minerals (i.e., magnetite, vivianite).

We deconvoluted the FTIR bands in specific spectral regions of the samples and references using a mixture of Gaussian and Lorentzian functions (G:L ratio ≥ 0.7), ensuring that the fewest components were used. The deconvoluted FTIR bands are presented in Figures S3 and S4, and the details resulting in fitted component bands are presented in Tables S5 and S10. It must be noted, however, that the phosphate stretching [$v(PO_4)$] region overlaps with the strong and split band representing the sulfate vibration [$v_3(SO_4)$] of GR_{SO4} at ~1098 cm^{-1} while the arsenate stretching [$v(AsO_4)$] region coincides with its lattice Fe–OH vibration at ~777 cm^{-1}.

Using the fitting approach that we have developed in our previous work,[5] we fitted the samples where GR_{SO4} was the dominant Fe phase (i.e., P/As ratio of 1 collected after 1 and 7 d) with the 4 component bands of $v_3(SO_4)$ from GR_{SO4}. The band shape parameters (i.e., height and area ratios) were kept similar to the reference spectra of GR_{SO4} (see Figure S3C, adapted from Perez et al.[5]). We then added the minimum number of component bands attributed to phosphate species to avoid overfitting the dataset. Finally, we checked and adjusted the band shape parameters (i.e., FWHM, G:L ratio) of the $v_3(SO_4)$ component bands to verify if the values have drifted from the desired height and area ratios. We could selectively assign the specific component bands associated with phosphate species in all our experimental samples using this procedure. In the case of samples where GR was not the dominant phase, we deconvoluted the spectra but only fitting two components (~1130 and ~1100 cm^{-1}). Similarly, we employed a similar approach for the deconvolution of the $v(AsO_4)$ region – fitting the lattice Fe–OH vibration of the synthetic GR_{SO4} reference into 4 component bands at ca. 827, 772, 742, and 717 cm^{-1} (Figure S4B and Table S10). When fitting the sample spectra, the band shape parameters were kept comparable to the reference values while minimizing the number of component bands to prevent overfitting.

Text S4. Arsenic K-edge X-ray absorption spectroscopy (XAS)

Inside the glovebox, powdered solid samples were mixed with cellulose at ratios calculated using the XAFSmass software,[6] ground, and then pressed into 13-mm pellets using a mini hydraulic 2-ton press (Specac Ltd.). The pressed pellets were sealed inside two layers of single-sided 70-μm thick Kapton® polyimide tape and placed inside air-tight headspace crimp vials stored in an anaerobic jar, preventing oxidation during transport to the beamline. Upon arrival at Diamond Light Source, sample vials were immediately transferred to the glovebox (Ar atmosphere, MBRAUN) and only removed before mounting in the I20 beamline cryostat system.

Arsenic K-edge XAS spectra were collected at the I20-scanning beamline of Diamond Light Source.[7] The vertical dimension of the X-ray beam during data collection was 400 μm, and the horizontal dimension was 300 μm. Rejection mirrors were used to prevent second-order harmonics. A Si(111) crystal pair ($\Delta E/E = 1.3\times10^{-4}$) with a fixed beam exit was used as a monochromator. The maximum in the first derivative of X-ray absorption spectra from an Au(0) foil was used to calibrate the beam at 11,919 eV. Spectra were aligned, averaged, and background subtracted using the Athena software.[8] Linear combination fitting (LCF) of the X-ray absorption near-edge spectra (XANES) was performed using reference standards (i.e., As(III) and As(V) adsorbed onto FHY) to determine As oxidation states in the solids. Meanwhile, the k^3-weighted extended X-ray absorption fine structure (EXAFS) spectra were extracted and Fourier-transformed over the k-range of 2 to 12.5 Å^{-1} using a Kaiser-Bessel window with a dk of 1 Å^{-1}, and succeeding shell-by-shell fits were performed from 1 to 4 Å in R+ΔR-space using SIXPack software[9] based on algorithms derived from IFEFFIT.[10]

The shell-by-shell fitting procedure included the following fitting parameters: interatomic distance (R), coordination number (CN), the mean squared atomic displacement parameter (σ^2), and the change in threshold energy (ΔE_0). Phase and amplitude functions for single and multiple scattering paths were calculated using FEFF6.[11] These included As-O, As-O-O, and As-Fe paths derived from the structure of scorodite,[12] which were then used for fitting the parameters of various As local bonding environments. The CN and σ^2 parameters correlated highly in the initial fits, leading to high fit-derived standard errors. Therefore, following previous work,[13,14] we constrained the σ^2 values in the second shell fits (As-Fe path) to values used in our earlier works[4,5,15] to reduce the high correlations. Consistent with previous studies,[14,16] the passive electron reduction parameter, S_0^2, was set to 1.0 in each fit. The goodness-of-fit was assessed based on the R-factor, defined as the mean square difference between the fit and the data on a point-by-point basis: R-factor = $\sum_i (\mathrm{data}_i - \mathrm{fit}_i)^2 / \sum_i (\mathrm{data}_i)^2$. An R-factor <0.05 is considered to reflect a reasonable fit.[17] The validity of the addition of all scattering paths was evaluated using the F-test for EXAFS as suggested by Downward et al.[18] This F-test, which has been applied in many previous studies[5,19,20] was used to assess whether the inclusion of an additional path (i.e., atomic correlation) would statistically improve a fit.

It is important to note that LCF was not applied to the As K-edge EXAFS dataset due to the high degree of similarity in the EXAFS oscillations among the reference compounds, except As(V)-incorporated magnetite (MGT). Given that LCF is a purely mathematical fitting method, such spectral similarity could lead to unreliable estimates of arsenic partitioning among the phases. Therefore, to prioritize structural interpretation over semi-quantitative estimation, we based our assessment of the dominant As-hosting phase on shell-by-shell fitting of the EXAFS spectra.

Supporting Figures

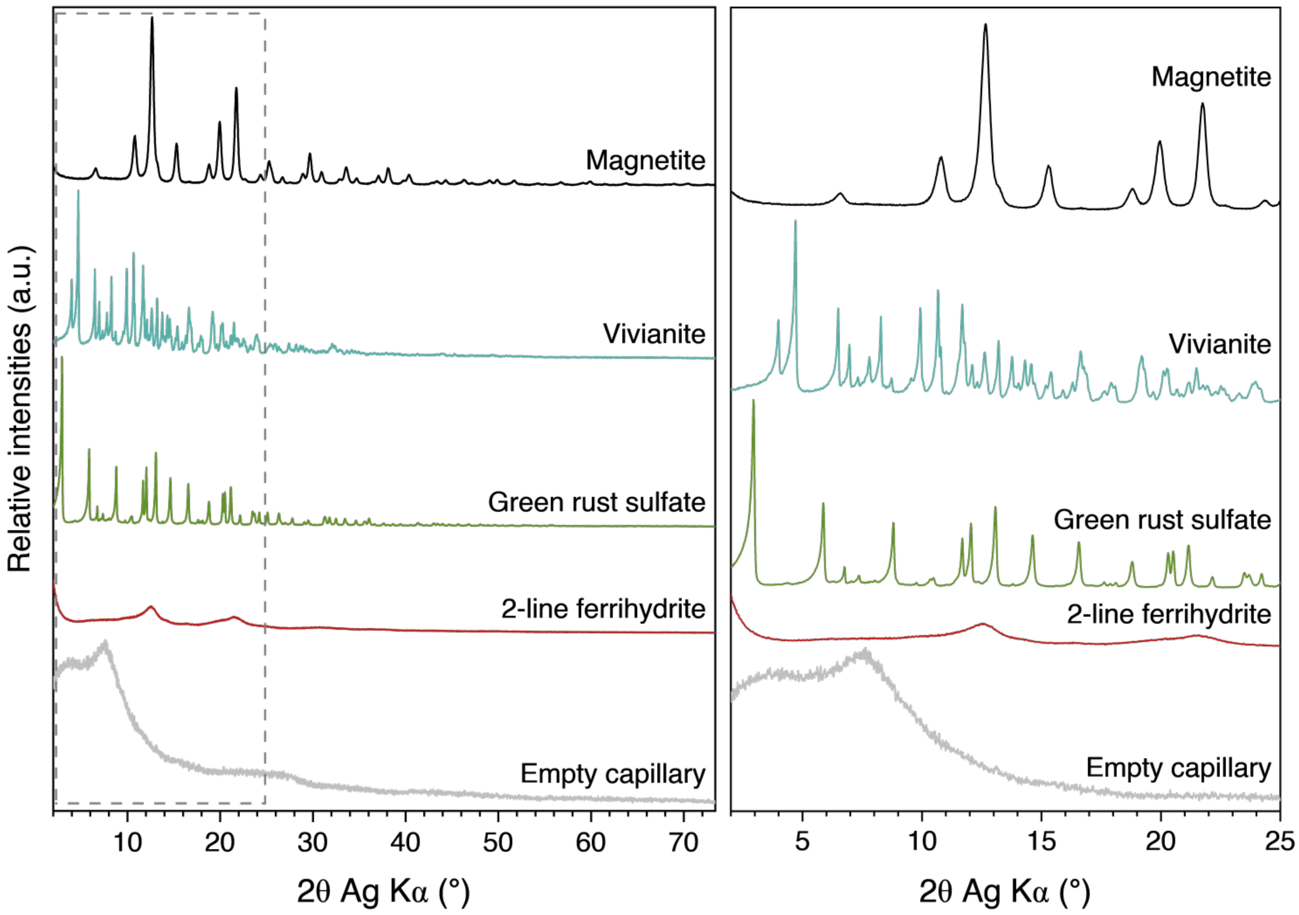

Figure S1. Powder XRD patterns of the synthesized reference Fe minerals (including empty capillary background). These reference phases were used for the Reverse Monte Carlo (RMC) method to estimate the solid phase composition in the produced experimental solids.

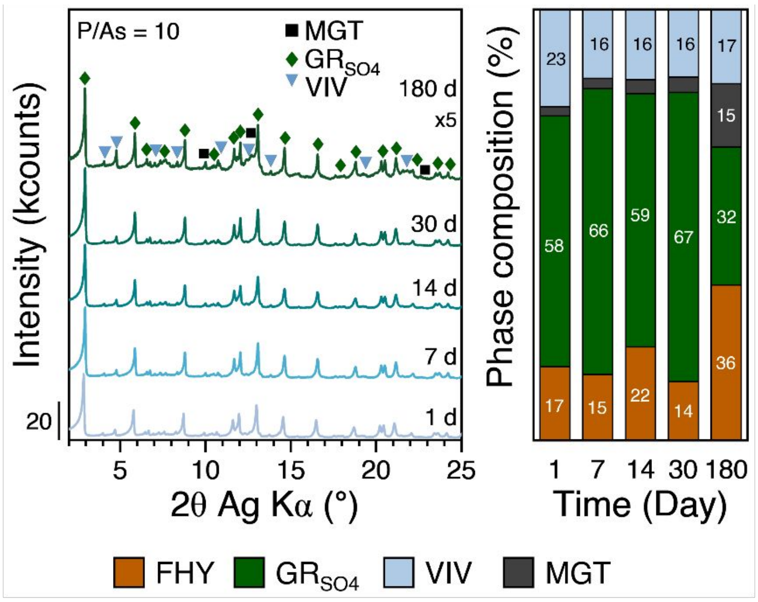

Figure S2. Powder XRD patterns and relative phase composition of solids collected at different elapsed times during the Fe^{2+}-induced transformation of FHY at varying P/As ratios of 10 ($[P]_{initial}$ = 1 mM). Bragg reflections are labeled to indicate crystalline Fe mineral phases: magnetite (MGT, black filled squares ■); GR_{SO4} (green filled diamonds ◆); and vivianite (VIV, light blue inverted triangles ▼).

Phases present below 5% relative proportion were not labeled.

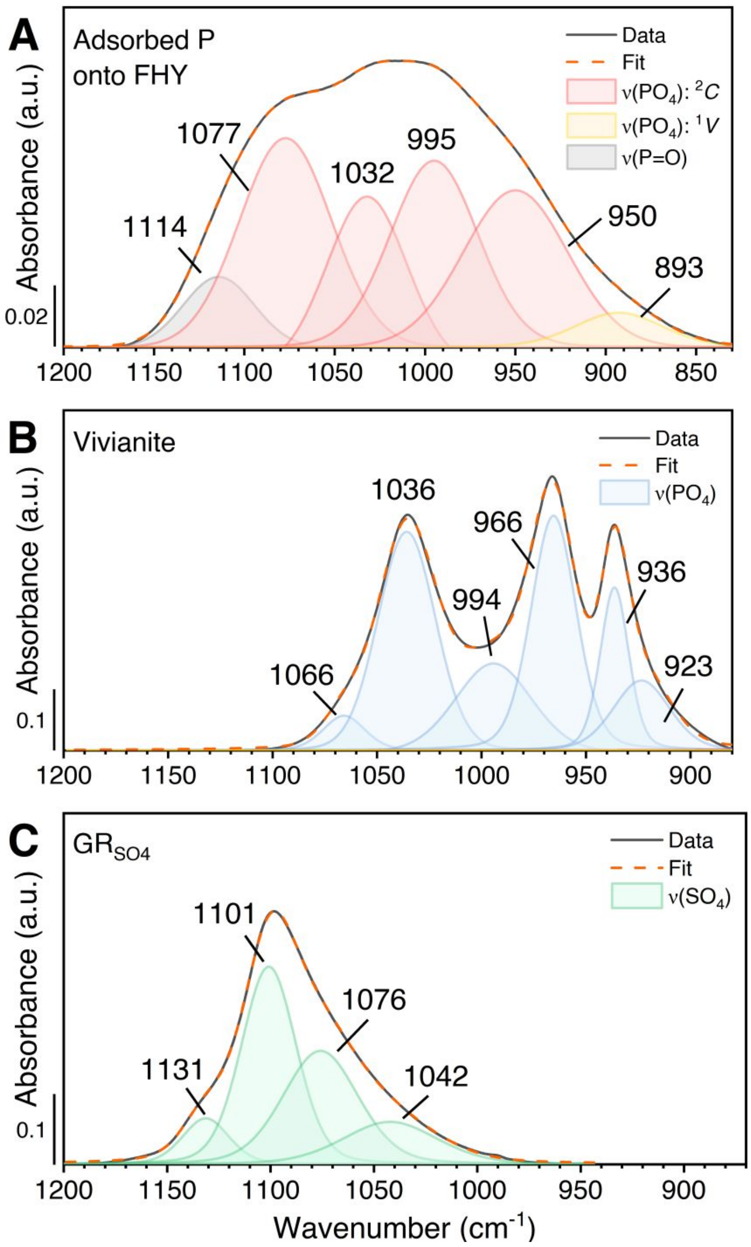

Figure S3. Deconvoluted FTIR spectra of synthesized reference phases used for band assignments in the phosphate region (1200 to 880 cm^{-1}): (a) adsorbed phosphate onto FHY; (b) synthetic vivianite;[15] and (c) synthetic GR_{SO4}.[5] The total fit (orange dashed lines) is superimposed on the experimental data (black solid line), and the fitting results and further statistics are given in Tables S5.

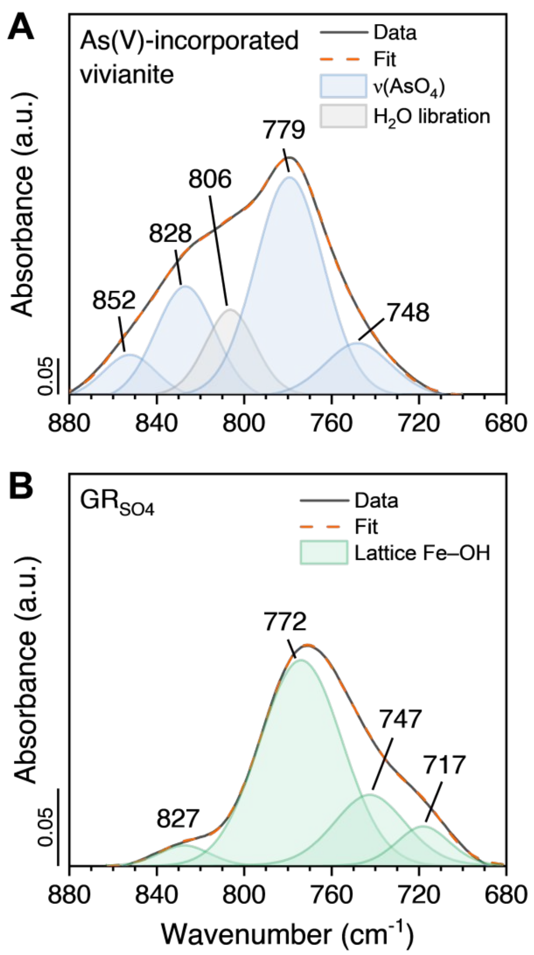

Figure S4. Deconvoluted FTIR spectra of synthesized reference phases used for band assignments in the arsenate region (880 to 680 cm^{-1}): (a) As(V)-incorporated vivianite;[15] and (b) synthetic GR_{SO4}.[5] The total fit (orange dashed lines) is superimposed on the experimental data (black solid line), and the fitting results and further statistics are given in Tables S10.

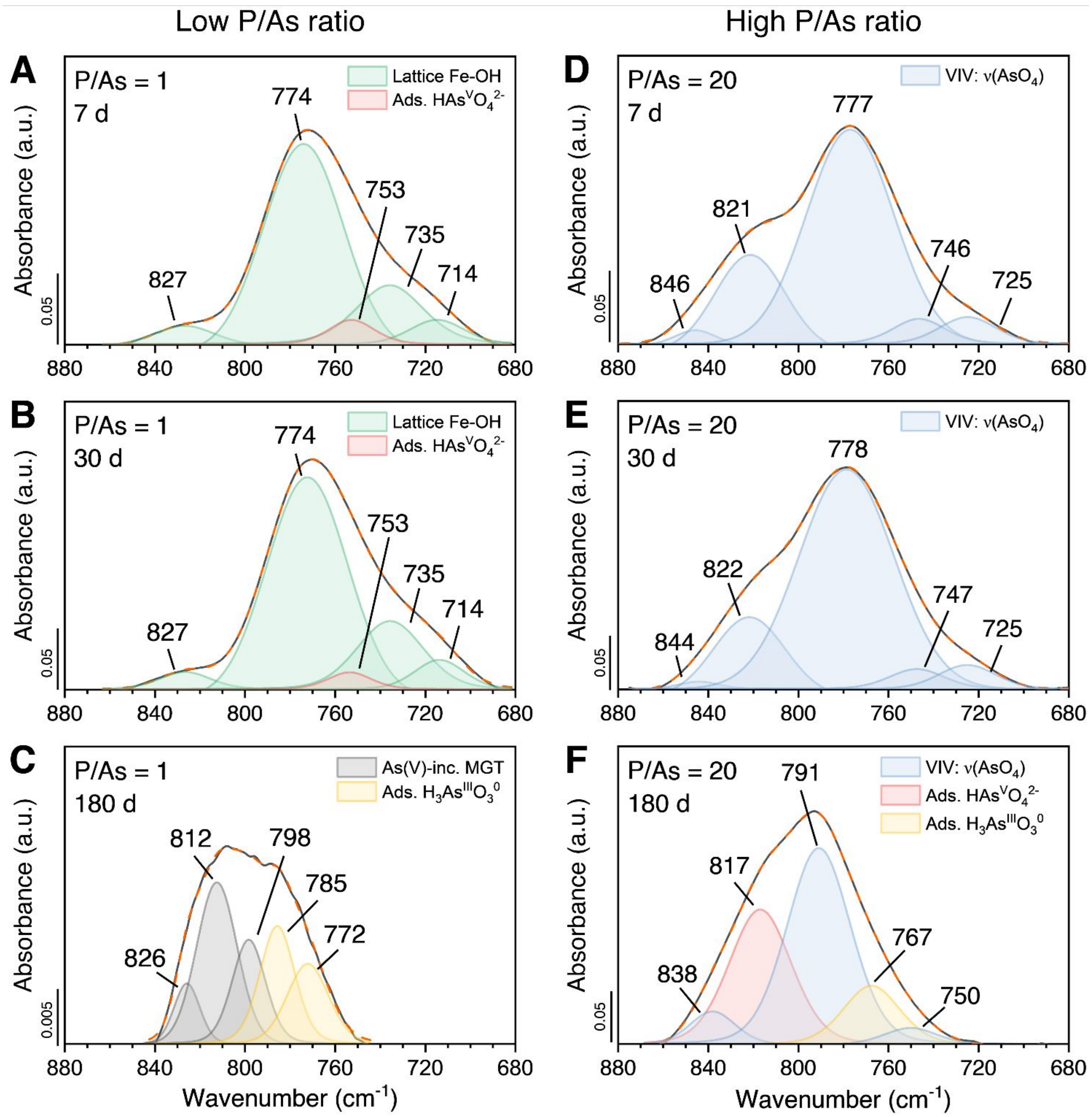

Figure S5. Deconvoluted FTIR spectra [ν(AsO_4) region: 880–680 cm^{-1}] of solids collected at different elapsed times at P/As ratios of (A-C) 1 and (D-F) 20. The total fit (orange dashed lines) is superimposed on the experimental data (black solid line), and the fitting results and further statistics are given in Tables S12 and S13.

Supplementary Tables

Table S1. Batch studies on As–and/or P–bearing metastable Fe(III) phase transformations under anoxic and circum-neutral pH conditions.

Fe(III) source	pH	$[Fe(III)]_{init}$ (mM)	Fe(II)/Fe(III) ratio	P/Fe(II) ratio	As/Fe_{tot} ratio	P/As_{tot} ratio	Aging time (d)	As(V) reduced?	Crystalline Fe phase(s)	Reference
P only										
$Fe^{3+}_{(aq)}$[a]	7.7	6	2	0.007–0.033	-	-	1	-	GR_{SO4}	Xiong et al.[21]
				0.167–0.333	-	-		-	GR_{SO4}, VIV	
				0.667	-	-		-	VIV	
FHY	7.0	4.4	3	0.19	-	-	-	-	GR_{SO4}, VIV	Hansen et al.[22]
Si-FHY[b]	7.0	50	0.55–0.70	0.02–0.08	-	-	1	-	GR_{CO3}	Kukkadapu et al.[23]
					-	-	450	-	GR_{CO3}	
				0.2	-	-	1	-	GR_{CO3}, VIV	
				0.4	-	-	1	-	VIV	
As(V) only										
Fe(0)[c]	8.0	-	2	-	0.02	-	7	No	GR_{CO3}	van Genuchten et al.[24]
						-	360	Yes	MGT	
LP	6.6	6	0.5	-	0.008	-	1	No	MGT	Wang et al.[25]
					0.04	-	1	No	GR_{Cl}	
FHY	8.0	4	3	-	0.008	-	1	No	GR_{SO4}	Perez et al.[4]
							30	No	MGT	
As(V)+P										
LP	7.0	5–7	0.35	0.017	0.003	5.6	5	Yes	VIV, GOE	Muehe et al.[26]
			0.53		0.015	1.1	5	Yes	SID, VIV, GOE	
FHY	7.0	4	2	0.009	0.009	1.2	7	No	GR_{SO4}, MGT	This study
							30	No	GR_{SO4}, MGT	
							180	Yes	MGT, GR_{SO4}	
				0.096–0.174		10.3 – 22.0	7	No	GR_{SO4}, VIV, MGT	
							30	Yes	GR_{SO4}, VIV, MGT	
							180	Yes	GR_{SO4}, VIV, MGT	

Note: FHY – ferrihydrite, GOE – goethite, GR – green rust, LP – lepidocrocite, magnetite – magnetite, SID – siderite, Si-FHY – silica-bearing FHY, VIV – vivianite. Unless otherwise indicated, the metastable Fe(III) precursors were transformed via Fe^{2+}-catalyzed recrystallization: [a]co-precipitation with aqueous Fe^{2+} and Fe^{3+}; [b]bioreduction of Si-FHY by dissimilatory iron-reducing bacteria (*Shewanella putrefaciens* strain CN32); and [c]Fe(0) electrolysis method.

Table S2. ICP-OES data for quality control solutions (QC) were prepared from single-element standard solutions (Merck, CertiPur) to achieve chemical compositions similar to the experimental sample solutions. The mean results of *n* replicate analyses are given together with the standard deviation (SD) and relative standard deviation (RSD) (SD represents 68% of the population, 2SD represents 95% of the population). The measured deviation from the reference value is a quantitative estimation of accuracy.

	**As (μg g^{-1})**	**P (μg g^{-1})**	**Fe (μg g^{-1})**
Wavelength (nm)	193.696	177.434	261.382
Instrumental limits (*n* = 12)			
Limit of detection (LOD)	0.010	0.018	0.004
Limit of quantification (LOQ)	0.039	0.048	0.007
Analytical session no. 1 (Samples from D1-D14)			
Quality control (*n* = 4)			
Mean measured value	0.311	2.170	4.173
SD	0.014	0.063	0.038
RSD	4.40%	2.89%	0.91%
2RSD	8.80%	5.79%	1.82%
Reference value	0.310	2.04	4.08
Uncertainty	0.005	0.005	0.010
Measured deviation from reference value	0.39%	6.21%	2.35%
Analytical session no. 2 (Samples from D30-D180)			
Quality control (n = 4)			
Mean measured value	0.325	2.188	3.920
SD	0.035	0.079	0.276
RSD	10.7%	3.62%	7.05%
2RSD	21.4%	7.24%	14.1%
Reference value	0.310	2.04	4.08
Uncertainty	0.005	0.005	0.010
Measured deviation from reference value	5.01%	7.10%	3.84%

Note: Liquid and digested suspension samples collected from 1 d to 14 d were analyzed in analytical session no. 1. The remaining samples (i.e., 30 d to 180 d) were measured in analytical session no. 2.

Table S3. Standard Gibbs free energy of formation ($\Delta G_f°$) at 25 °C.

	$\Delta \boldsymbol{G_f}°$ **(kJ mol^{-1})**	**Reference**
Minerals		
Vivianite [$Fe^{II}_3(PO_4)_2 \cdot 8H_2O$]	-4,439.0	Ogorodova et al.[27]
Green rust sulfate [$Fe^{II}_4Fe^{III}_2(OH)_{12}SO_4$]	-3,819.4	Ayala-Luis et al.[28]
Magnetite [$Fe^{II}Fe^{III}_2O_4$]	-1,012.7	Robie et al.[29]
Maghemite [γ-$Fe^{III}_2O_3$]	-727.9	Majzlan et al.[30]
2-line ferrihydrite [$Fe^{III}(OH)_3$]	-708.5	Majzlan et al.[31]
Aqueous species		
Fe^{2+}	-90.0	Robie et al.[29]
H_2O	-237.1	Robie et al.[29]
$As^{III}(OH)_3$	-639.9	Nordstrom et al.[32]
$H_2As^{V}O_4^-$	-753.6	Nordstrom et al.[32]
$HAs^{V}O_4^{2-}$	-713.7	Nordstrom et al.[32]
$H_2PO_4^-$	-1137.2	Rard et al.[33]
HPO_4^{2-}	-1096.0	Rard et al.[33]
SO_4^{2-}	-744.0	Nordstrom et al.[32]

Table S4. Changes in aqueous [As], [P], and [Fe^{2+}] concentrations during the Fe^{2+}-induced transformation of As/P-bearing ferrihydrite at varying P/As ratios. Values in parenthesis denote the corresponding removal efficiencies (%) relative to the initial concentration.

P/As ratio	Fe^{2+} [mM]						P [µM]						As [µM]					
	**0 d**	**1 d**	**7 d**	**14 d**	**30 d**	**180 d**	**0 d**	**1 d**	**7 d**	**14 d**	**30 d**	**180 d**	**0 d**	**1 d**	**7 d**	**14 d**	**30 d**	**180 d**
0	8.66	1.95 (78)	2.19 (75)	2.26 (74)	2.53 (71)	2.94 (66)	-	-	-	-	-	-	-	-	-	-	-	-
1	8.97	2.10 (77)	2.11 (77)	2.15 (76)	2.11 (76)	2.95 (67)	177	1.25 (99)	0.68 (>99)	1.11 (>99)	0.56 (>99)	0.62 (>99)	107	0.29 (>99)	0.18 (>99)	0.16 >99)	0.40 (>99)	0.33 (>99)
5	8.88	1.39 (84)	1.35 (85)	1.36 (85)	1.35 (85)	1.70 (81)	695	8.45 (99)	4.09 (99)	3.52 (>99)	0.97 (>99)	0.68 (>99)	106	0.71 (99.3)	0.30 (>99)	0.16 (>99)	0.53 (>99)	0.53 (>99)
10	8.85	1.39 (84)	1.40 (84)	1.44 (84)	1.42 (84)	1.89 (79)	1355	5.44 (>99)	3.53 (>99)	6.78 (>99)	2.18 (>99)	1.67 (>99)	102	0.28 (>99)	0.16 (>99)	0.38 (>99)	0.64 (99)	0.51 (>99)
20	8.86	0.57 (94)	0.53 (94)	0.61 (93)	0.65 (93)	1.05 (88)	2733	5.96 (>99)	0.68 (>99)	4.17 (>99)	2.64 (>99)	1.66 (>99)	105	0.19 (>99)	0.11 (>99)	0.06 (>99)	0.64 (99)	0.26 (>99)

Table S5. Details of the deconvolution of FTIR spectra (1200 to 880 cm^{-1}) of phosphate reference phases, (i) P adsorbed onto FHY; (ii) PO_4 bands in vivianite; and (iii) SO_4 bands in GR_{SO4}.

No.	Position (cm^{-1})	Height (a.u.)	FWHH (cm^{-1})	G:L ratio	Area (a.u.)	Assignment	Species	References
Adsorbed P onto FHY								
1	1114	0.023	46	0.79	1.12	v(P=O)	-	
2	1077	0.068	60	0.97	4.38		≡Fe_2PO_4, ^2C complex	
3	1032	0.056	53	0.92	3.27	$v_{as}(PO_4)$	≡Fe_2PO_4, ^2C complex	Elzinga et al.[34], Arai et al.[35]
4	995	0.060	57	0.92	3.83		≡Fe_2PO_4H, ^2C complex	
5	945	0.051	68	0.98	3.75		≡Fe_2PO_4, ^2C complex	
6	892	0.011	58	1.00	0.76	$v_s(PO_4)$	≡$FePO_4H$, ^1V complex	
Synthetic Vivianite								
1	1066	0.053	23	0.72	1.45			
2	1036	0.355	33	0.87	13.18			
3	994	0.140	42	0.76	6.99	$v(PO_4)$	PO_4 tetrahedra in the crystal structure	Perez et al.[15], Capitelli et al.[36], Frost et al.[37], Martens et al.[38]
4	966	0.385	26	0.74	11.89			
5	936	0.271	16	0.72	5.32			
6	923	0.108	30	0.74	3.90			
Synthetic GR_{SO4}								
1	1131	0.069	28	0.76	2.25			
2	1101	0.264	30	0.83	9.22	$v(SO_4)$	SO_4 tetrahedra in the interlayer space	Perez et al.[5], Peulon et al.[39]
3	1078	0.171	45	0.82	8.81			
4	1042	0.061	55	0.75	4.01			

Note: FWHH denotes full width at half height, and the G:L ratio refers to the Gaussian/Lorentzian ratio used for the fitting.

Table S6. Deconvolution of FTIR spectra in the range between 1200 and 880 cm^{-1} for the As+P–bearing solids separated from the reacting solutions at different elapsed times during the Fe^{2+}-induced transformation of FHY at a P/As ratio of 1 at 7, 30 and 180 d. See Fig. 2B in the main text for relative phase composition.

No.	Position (cm^{-1})	Height (a.u.)	FWHH (cm^{-1})	G:L ratio	Area (a.u.)	Assignment	Mineral phase or speciation
P/As = 1, 7 d							
1	1131	0.067	25	0.92	1.84	v(SO_4)	GR_{SO4}
2	1102	0.325	31	0.33	14.32	v(SO_4)	GR_{SO4}
3	1082	0.136	41	0.69	6.73	v(SO_4)	GR_{SO4}
4	1061	0.051	35	0.75	2.11	v(PO_4)	^{2}C complex
5	1039	0.064	43	0.78	3.27	v(SO_4)	GR_{SO4}
6	1014	0.014	32	0.76	0.55	v(PO_4)	^{2}C complex
7	987	0.012	29	0.90	0.39	v(PO_4)	^{2}C complex
8	896	0.014	37	0.84	0.58	Lattice Fe-OH	GR_{SO4}
P/As = 1, 30 d							
1	1130	0.08	24	0.91	2.07	v(SO_4)	GR_{SO4}
2	1100	0.40	31	0.34	17.38	v(SO_4)	GR_{SO4}
3	1082	0.21	41	0.69	10.60	v(SO_4)	GR_{SO4}
4	1061	0.08	35	0.75	3.17	v(PO_4)	^{2}C complex
5	1040	0.09	43	0.78	4.82	v(SO_4)	GR_{SO4}
6	1013	0.02	33	0.76	0.72	v(PO_4)	^{2}C complex
7	987	0.02	29	0.89	0.61	v(PO_4)	^{2}C complex
8	896	0.02	38	0.84	0.65	Lattice Fe-OH	GR_{SO4}
P/As = 1, 180 d							
1	1156	0.179	32	1.00	5.99	v(P=O)	–
2	1130	0.062	26	0.75	1.88	v(SO_4)	GR_{SO4}
3	1105	0.103	39	0.82	4.58	v(SO_4)	GR_{SO4}
4	1079	0.241	44	0.80	12.44	v(PO_4)	^{2}C complex
5	1046	0.170	46	0.76	9.17	v(PO_4)	Vivianite
6	1015	0.108	41	0.75	5.28	v(PO_4)	^{2}C complex
7	985	0.067	31	0.65	2.54	v(PO_4)	Vivianite
8	959	0.085	43	0.83	4.18	v(PO_4)	^{2}C complex
9	925	0.024	30	0.71	0.87	v(PO_4)	Vivianite

Note: FWHH denotes full width at half height, and the G:L ratio refers to the Gaussian/Lorentzian ratio used for the fitting.

Table S7. Deconvolution of FTIR spectra in the range between 1200 and 880 cm^{-1} for the As+P–bearing solids separated from the reacting solutions at different elapsed times during the Fe^{2+}-induced transformation of FHY at a P/As ratio of 20 at 7, 30 and 180 d. See Fig. 2D in the main text for relative phase composition.

No.	Position (cm^{-1})	Height (a.u.)	FWHH (cm^{-1})	G:L ratio	Area (a.u.)	Assignment	Mineral phase or speciation
P/As = 20, 7 d							
1	1133	0.068	23	0.85	1.75	$v(SO_4)$	GR_{SO4}
2	1103	0.264	31	0.39	11.02	$v(SO_4)$	GR_{SO4}
3	1059	0.120	41	0.70	5.89	$v(PO_4)$	^{2}C complex
4	1035	0.172	26	0.61	5.54	$v(PO_4)$	Vivianite
5	1014	0.038	30	0.67	1.41	$v(PO_4)$	^{2}C complex
6	984	0.038	25	0.68	1.17	$v(PO_4)$	^{2}C complex
7	967	0.181	21	0.66	4.62	$v(PO_4)$	Vivianite
8	937	0.106	14	0.65	1.80	$v(PO_4)$	Vivianite
9	924	0.014	17	0.71	0.29	v(P-OH)	–
P/As = 20, 30 d							
1	1132	0.075	22	0.82	1.93	$v(SO_4)$	GR_{SO4}
2	1103	0.277	30	0.42	11.22	$v(SO_4)$	GR_{SO4}
3	1057	0.135	39	0.56	6.81	$v(PO_4)$	^{2}C complex
4	1035	0.231	25	0.60	7.22	$v(PO_4)$	Vivianite
5	1015	0.027	28	0.67	0.94	$v(PO_4)$	^{2}C complex
6	978	0.065	23	0.68	1.87	$v(PO_4)$	^{2}C complex
7	967	0.199	18	0.74	4.20	$v(PO_4)$	Vivianite
8	937	0.120	12	0.77	1.76	$v(PO_4)$	Vivianite
9	894	0.016	20	0.66	0.40	$v(PO_4)$	^{1}V complex
P/As = 20, 180 d							
1	1164	0.013	28	0.75	0.44	v(P=O)	–
2	1134	0.038	35	0.78	1.57	$v(SO_4)$	GR_{SO4}
3	1106	0.055	38	0.76	2.46	$v(SO_4)$	GR_{SO4}
4	1060	0.089	30	0.72	3.25	$v(PO_4)$	^{2}C complex
5	1035	0.220	27	0.57	7.73	$v(PO_4)$	Vivianite
6	1013	0.022	28	0.51	0.79	$v(PO_4)$	^{2}C complex
7	982	0.042	23	0.70	1.16	$v(PO_4)$	^{2}C complex
8	967	0.202	20	0.71	4.87	$v(PO_4)$	Vivianite
9	936	0.119	14	0.73	1.97	$v(PO_4)$	Vivianite
10	887	0.013	20	0.73	0.33	$v(PO_4)$	^{1}V complex

Note: FWHH denotes full width at half height, and the G:L ratio refers to the Gaussian/Lorentzian ratio used for the fitting.

Table S8. As K-edge XANES linear combination fit results and statistics of the solid phases collected at different elapsed times in the experiments conducted at P/As ratios of 1 and 20 for between 7 and 180 days. Values in parentheses denote analytical uncertainty.

Sample	Component weights		Sum	Red. X^2	R-factor	Bulk composition (%)	
	As(III)	As(V)				As(III)	As(V)
P/As = 1<br>7 d	-	0.997<br>(0.010)	0.997<br>(0.010)	0.001	0.001	-	100
P/As = 1<br>30 d	-	0.996<br>(0.009)	0.996<br>(0.009)	0.001	0.001	-	100
P/As = 1<br>180 d	0.152<br>(0.006)	0.852<br>(0.007)	1.004<br>(0.009)	0.001	0.002	15.2<br>(0.6)	84.9<br>(1.0)
P/As = 20<br>7 d	-	1.012<br>(0.004)	1.012<br>(0.004)	0.001	0.001	-	100
P/As = 20<br>30 d	0.069<br>(0.026)	0.944<br>(0.021)	1.013<br>(0.033)	0.001	0.001	6.8<br>(2.6)	93.2<br>(3.7)
P/As = 20<br>180 d	0.259<br>(0.014)	0.751<br>(0.016)	1.010<br>(0.021)	0.001	0.001	25.6<br>(1.1)	74.4<br>(2.7)

Table S9. Summary of the As K-edge EXAFS shell-by-shell fits of mineral solids collected at different elapsed times at P/As ratios of 1 and 20, and As reference phases. Values in parentheses denote analytical uncertainty.

Sample	Atomic pairs	*CN*	*R* (Å)	σ^2 (Å^2)	ΔE_0 (eV)	*R*-factor
P/As = 1 7 d	As-O	4.6 (0.4)	1.69 (0.01)	0.003 (0.001)	3.9 (1.3)	0.023
	As-O-O	12	1.82(R_{As-O}) = 3.07	σ^2 (As-O)		
	As-Fe	3.4 (0.7)	3.38 (0.02)	*0.009*		
P/As = 1 30 d	As-O	4.7 (0.4)	1.69 (0.01)	0.003 (0.001)	3.5 (1.3)	0.023
	As-O-O	12	1.82(R_{As-O}) = 3.07	σ^2 (As-O)		
	As-Fe	3.2 (0.7)	3.40 (0.02)	*0.009*		
P/As = 1 180 d	As-O	4.3 (0.4)	1.71 (0.01)	0.004 (0.001)	4.0 (1.3)	0.022
	As-O-O	12	1.82(R_{As-O}) = 3.10	σ^2 (As-O)		
	As-Fe1	8.8 (1.3)	3.47 (0.01)	*0.009*		
	As-Fe2	4.5 (1.3)	3.63 (0.03)	σ^2 (As-Fe1)		
P/As = 20 7 d	As-O	4.9 (0.5)	1.69 (0.01)	0.005 (0.001)	4.1 (1.5)	0.027
	As-O-O	12	1.82(R_{As-O}) = 3.08	σ^2 (As-O)		
	As-Fe1	4.0 (1.2)	3.30 (0.02)	*0.009*		
	As-Fe2	3.0 (1.4)	3.47 (0.04)	σ^2 (As-Fe1)		
P/As = 20 30 d	As-O	4.1 (0.4)	1.70 (0.01)	0.004 (0.001)	3.8 (1.3)	0.018
	As-O-O	12	1.82(R_{As-O}) = 3.09	σ^2 (As-O)		
	As-Fe1	3.0 (0.9)	3.28 (0.03)	*0.009*		
	As-Fe	2.9 (1.0)	3.43 (0.03)	σ^2 (As-Fe1)		
P/As = 20 180 d	As-O	3.9 (0.5)	1.72 (0.01)	0.004 (0.001)	7.7 (1.9)	0.049
	As-O-O	12	1.82(R_{As-O}) = 3.14	σ^2 (As-O)		
	As-Fe1	1.4 (1.3)	3.25 (0.06)	*0.009*		
	As-Fe2	3.5 (1.5)	3.43 (0.03)	σ^2 (As-Fe1)		
References						
As(V)-cop. FHY	As-O	4.6 (0.4)	1.69 (0.01)	0.003 (0.001)	5.0 (1.3)	0.022
	As-O-O	12	1.82(R_{As-O}) = 3.08	σ^2 (As-O)		
	As-Fe	2.3 (0.7)	3.30 (0.02)	*0.01*		
As(V)-cop. GR_{SO4}	As-O	4.6 (0.4)	1.69 (0.01)	0.003 (0.001)	4.8 (1.3)	0.021
	As-O-O	12	1.82(R_{As-O}) = 3.07	σ^2 (As-O)		
	As-Fe	1.8 (0.7)	3.41 (0.02)	*0.009*		
As(V)-cop. MGT	As-O	4.6 (0.4)	1.70 (0.01)	0.003 (0.001)	4.5 (1.2)	0.019
	As-O-O	12	1.82(R_{As-O}) = 3.09	σ^2 (As-O)		
	As-Fe1	7.2 (1.2)	3.46 (0.01)	*0.009*		
	As-Fe2	3.4 (1.5)	3.65 (0.03)	σ^2 (As-Fe1)		
As(V)-cop. VIV	As-O	4.3 (0.5)	1.69 (0.01)	0.005 (0.001)	4.7 (1.6)	0.033
	As-O-O	12	1.82(R_{As-O}) = 3.08	σ^2 (As-O)		
	As-Fe1	2.9 (0.7)	3.29 (0.02)	*0.006*		
	As-Fe2	1.8 (0.6)	3.46 (0.02)	*0.004*		

Note: CN, coordination number; *R*, interatomic distance; σ^2, mean-squared atomic displacement; and ΔE_0, change in threshold energy. The passive electron reduction factor (S_0^2) was fixed at 1.0. The multiple scattering As-O-O path was constrained geometrically to the single scattering As-O path ($R_{As\text{-}O\text{-}O}$ = 1.82 × $R_{As\text{-}O}$). All fits were carried out from 1 to 3.5 Å in R+ΔR - space. The number of independent points (N_{IDP}) in the fits was 19.8, and the number of variables (NVar) was between 6 and 8.

Table S10. Details of the deconvolution of FTIR spectra (880 to 680 cm^{-1}) of phosphate reference phases: (i) As(V)-substituted VIV; and (ii) synthetic GR_{SO4}.

No.	Position (cm^{-1})	Height (a.u.)	FWHH (cm^{-1})	G:L ratio	Area (a.u.)	Vibrational mode	Assignment	References
Synthetic As(V)-incorporated vivianite (48 mol% substitution)								
1	852	0.057	29	0.85	1.92	v(As–O)	As(V)-incorporated vivianite	Perez et al.[15], Frost et al.[40], Myneni et al.[41], Makreski et al.[42]
2	828	0.151	33	0.86	5.60	v(As–O)		
3	806	0.115	27	0.77	3.71	H_2O librational mode		
4	779	0.287	37	0.92	11.74	v(As–O–Fe)		
5	748	0.071	37	0.80	3.02	v(As–O)		
Synthetic GR_{SO4}								
1	827	0.014	26	0.72	0.43	Lattice Fe–OH	Octahedrally coordinated Fe(II)/Fe(III)–OH	Perez et al.[5], Peulon et al.[39]
2	772	0.180	42	0.99	8.12			
3	742	0.058	39	0.80	2.62			
4	717	0.031	29	0.77	1.05			

Note: FWHH denotes full width at half height, and the G:L ratio refers to the Gaussian/Lorentzian ratio used for the fitting.

Table S11. Additional FTIR spectra references used for band assignment of fitted component bands resulting from the deconvolution of arsenate region (880-680 cm^{-1}).

Centroid position	Vibrational mode	Assignment	References
Adsorbed As(III) species on FHY			
875–882	v(As–O–Fe)	Adsorbed $H_3As^{III}O_3^0$ in bidentate, binuclear (^2C) geometry	Müller et al.[43], Zheng et al.[44]
782–791	v(As–O–Fe)	Adsorbed $H_3As^{III}O_3^0$ in monodentate, mononuclear (^1V) geometry	Goldberg et al.[45], Müller et al.[43], Zheng et al.[44]
770–774	$v(AsO_3)$	Adsorbed $H_2As^{III}O_3^-$ or $H_3As^{III}O_3^0$	Voegelin et al.[46], Bhandari et al.[47]
As(III)-bearing mineral phases			
773	$v_s(AsO_3)$	Tooelite,	Liu et al.[48]
734	$v_{as}(AsO_3)$	$Fe_6(As^{III}O_3)_4SO_4(OH)_4{\cdot}4H_2O$	
Adsorbed As(V) species on FHY			
870–878	v(As–O)	Non-surface complexed As–O or As–O–Fe	Myneni et al.[41], Jia et al.[49], Li et al.[50]
854–861	v(As–O)	Non-surface complexed As–O	Goldberg et al.[45], Voegelin et al.[46], Gao et al.[51]
817–824	v(As–O–Fe)	Adsorbed $HAs^VO_4^{2-}$ in ^2C geometry	Goldberg et al.[45]
810–819	v(As–O–Fe)	Adsorbed $HAs^VO_4^{2-}$ in ^2C geometry	Voegelin et al.[46], Li et al.[50]
800–808	v(As–O–Fe)	Adsorbed $HAs^VO_4^{2-}$ in ^2C geometry	Myneni et al.[41], Jia et al.[49], Müller et al.[43], Gao et al.[51]
750–765	v(As–O–X)	Complexed As–O–X (X = H^+, cations, H_2O)	Jia et al.[49], Li et al.[50]
700	v(As–O–H)	Protonated As–O–H bond in $HAsO_4^{2-}$	Myneni et al.[41], Jia et al.[49]
Adsorbed As(V) species on MGT			
870	v_{as}(As–O)		Gao et al.[51]
855		$HAsO_4^{2-}$	Gao et al.[51]
830	v_{as}(As–O–Fe)	Adsorbed $HAs^VO_4^{2-}$ in ^2C geometry	Gao et al.[51]
816	v(As–O–Fe)	Adsorbed $HAs^VO_4^2$	Zubair et al.[52]
805	v_s(As–O–Fe)	Adsorbed $HAs^VO_4^{2-}$ in ^2C geometry	Gao et al.[51]
As(V)-bearing mineral phases			
825–850	v_{as}(As–O)	Parasymplesite, $Fe^{II}_3(AsO_4)_2{\cdot}8H_2O$	Myneni et al.[41], Makreski et al.[42], Frost et al.[40]
768–795	v_{as}(As–O–Fe)		
736	v_s(As–O)		
690	H_2O librational mode		
900	v_{as}(As–O)	Scorodite, $FeAsO_4{\cdot}2H_2O$	Gomez et al.[53]
795	v_s(As–O)		
720	v_{as}(As–O–Fe)		

Table S12. Deconvolution of FTIR spectra in the range between 880 and 680 cm^{-1} for the As+P–bearing solids separated from the reacting solutions at different elapsed times during the Fe^{2+}-induced transformation of FHY at a P/As ratio of 1 at 7, 30 and 180 d. See Fig. 2B in the main text for relative phase composition.

No.	Centroid position (cm^{-1})	Height (a.u.)	FWHH (cm^{-1})	G:L ratio	Area (a.u.)	Vibrational mode	Band assignment
P/As = 1, 7 d							
1	827	0.015	33	0.70	0.59	Lattice Fe–OH	GR_{SO4}
2	774	0.145	42	1.00	6.40	Lattice Fe–OH	GR_{SO4}
3	753	0.018	25	0.69	0.54	v(As–O–H)	Adsorbed $HAs^{V}O_4^{2-}$
4	735	0.042	37	0.83	1.78	Lattice Fe–OH	GR_{SO4}
5	713	0.015	29	0.80	0.51	Lattice Fe–OH	GR_{SO4}
P/As = 1, 30 d							
1	826	0.015	27	0.74	0.47	Lattice Fe–OH	GR_{SO4}
2	772	0.184	42	1.00	8.27	Lattice Fe–OH	GR_{SO4}
3	753	0.014	23	0.69	0.40	v(As–O–H)	Adsorbed $HAs^{V}O_4^{2-}$
4	735	0.057	35	0.83	2.28	Lattice Fe–OH	GR_{SO4}
5	713	0.025	26	0.80	0.75	Lattice Fe–OH	GR_{SO4}
P/As = 1, 180 d							
1	826	0.005	14	0.85	0.08	v(As–O–Fe)	As(V)-inc. MGT
2	812	0.014	21	0.93	0.32	v(As–O–Fe)	As(V)-inc. MGT
3	798	0.009	18	0.78	0.18	v(As–O–Fe)	As(V)-inc. MGT
4	785	0.010	18	0.78	0.22	v(As–O–Fe)	Adsorbed $H_3As^{III}O_3^0$
5	772	0.007	22	0.81	0.17	v(As–O)	Adsorbed $H_3As^{III}O_3^0$

Note: FWHH denotes full width at half height, and the G:L ratio refers to the Gaussian/Lorentzian ratio used for the fitting.

Table S13. Deconvolution of FTIR spectra in the range between 880 and 680 cm^{-1} for the As+P–bearing solids separated from the reacting solutions at different elapsed times during the Fe^{2+}-induced transformation of FHY at a P/As ratio of 20 at 7, 30 and 180 d. See Fig. 2D in the main text for relative phase composition.

No.	Centroid position (cm^{-1})	Height (a.u.)	FWHH (cm^{-1})	G:L ratio	Area (a.u.)	Vibrational mode	Band assignment
P/As = 20, 7 d							
1	846	0.011	22	0.76	0.29	v(As–O)	As(V)-incorporated VIV
2	821	0.067	37	1.00	2.63	v(As–O)	
3	777	0.149	46	1.00	7.24	v(As–O–Fe)	
4	746	0.017	28	0.75	0.57	v(As–O)	
5	725	0.019	30	0.85	0.63	v(As–O–Fe)	
P/As = 20, 30 d							
1	844	0.007	21	0.76	0.17	v(As–O)	As(V)-incorporated VIV
2	822	0.075	37	1.00	2.97	v(As–O)	
3	778	0.206	50	1.00	10.88	v(As–O–Fe)	
4	747	0.019	29	0.75	0.64	v(As–O)	
5	725	0.023	30	0.86	0.76	v(As–O–Fe)	
P/As = 20, 180 d							
1	838	0.017	24	0.76	0.49	v(As–O)	As(V)-inc. VIV
2	817	0.068	33	0.80	2.60	v(As–O–Fe)	Adsorbed $HAs^{V}O_4^{2-}$
3	791	0.099	34	0.82	3.83	v(As–O–Fe)	As(V)-inc. VIV
4	767	0.030	30	0.74	1.07	v(As–O)	Adsorbed $H_3As^{III}O_3^0$
5	750	0.009	28	0.71	0.30	v(As–O)	As(V)-inc. VIV

Note: FWHH denotes full width at half height, and the G:L ratio refers to the Gaussian/Lorentzian ratio used for the fitting.

Table S14. Mineral saturation indices of GR_{SO4}, vivianite, parasymplesite, amorphous $Fe(OH)_2$, and crystalline $Fe(OH)_2$ (i.e., white rust) based on the elemental composition of the starting solutions used in the precipitation experiments.

P/As ratio	Concentration (mM)			Saturation indices					
	Fe^{2+}	HPO_4^{2-}	$HAsO_4^{2-}$	GR_{SO4}	Magnetite	Vivianite	Parasymplesite	$Fe(OH)_2$, am.	$Fe(OH)_2$, cryst.
0	8.66	-	-	20.6	28.7	-	-	-2.0	-1.4
1	8.97	0.18	0.11	20.6	28.7	9.5	7.4	-2.0	-1.4
5	8.88	0.70	0.11	20.5	28.7	10.6	7.3	-2.0	-1.4
10	8.85	1.35	0.10	20.4	28.6	11.2	7.1	-2.0	-1.4
20	8.86	2.73	0.11	20.1	28.6	11.7	7.0	-2.1	-1.5

Table S15. Calculated Gibbs free energies ($\Delta G_{rxn}°$)[a] at 25 °C of other candidate redox reactions involved in the partial reduction of As(V) to As(III).

Rxn no.	Chemical reaction	$\Delta G_{rxn}°$ (kJ mol^{-1})[a]
Vivianite		
1	$Fe^{II}_3(PO_4)_2 \cdot 8H_2O + 3HAs^{V}O_4^{2-} + 4H^+ \rightleftharpoons 3Fe^{III}(OH)_3 + 2H_2PO_4^- + 3As^{III}(OH)_3 + 2H_2O$	-209.5
2	$Fe^{II}_3(PO_4)_2 \cdot 8H_2O + 3HAs^{V}O_4^{2-} \rightleftharpoons 3Fe^{III}(OH)_3 + 2HPO_4^{2-} + 3As^{III}(OH)_3 + 2H_2O + 4H^+$	-131.3
Magnetite		
3	$Fe^{II}Fe^{III}_2O_4 + HAs^{V}O_4^{2-} + 4H_2O + 2H^+ \rightleftharpoons 3Fe^{III}(OH)_3 + 3As^{III}(OH)_3$	-90.6
4	$2Fe^{II}Fe^{III}_2O_4 + 2HAs^{V}O_4^{2-} + 4H^+ \rightleftharpoons 3\gamma\text{-}Fe^{III}_2O_3 + 2As^{III}(OH)_3 + H_2O$	-247.8
Green rust sulfate		
5	$Fe^{II}_4Fe^{III}_2(OH)_{12}SO_4 + 2HAs^{V}O_4^{2-} + 4H_2O + 2H^+ \rightleftharpoons 6Fe^{III}(OH)_3 + 2As^{III}(OH)_3 + SO_4^{2-}$	-79.6
6	$Fe^{II}_4Fe^{III}_2(OH)_{12}SO_4 + HAs^{V}O_4^{2-} \rightleftharpoons 2Fe^{II}Fe^{III}_2O_4 + As^{III}(OH)_3 + SO_4^{2-} + 5H_2O$	-61.7
Aqueous Fe^{2+}		
6	$2Fe^{2+} + HAs^{V}O_4^{2-} + 3H_2O \rightleftharpoons 2Fe^{III}(OH)_3 + As^{III}(OH)_3 + 2H^+$	22.3

Note: [a]Values were calculated from the standard Gibbs free energies ($\Delta G_f°$) of minerals and aqueous species (Table S3).

References

(1) Perez, J. P. H.; Freeman, H. M.; Schuessler, J. A.; Benning, L. G. The interfacial reactivity of arsenic species with green rust sulfate (GR_{SO4}). *Sci. Total Environ.* **2019**, *648*, 1161-1170.

(2) Paskin, A.; Couasnon, T.; Perez, J. P. H.; Lobanov, S. S.; Blukis, R.; Reinsch, S.; Benning, L. G. Nucleation and crystallization of ferrous phosphate hydrate via an amorphous intermediate. *J. Am. Chem. Soc.* **2023**, *145* (28), 15137-15151.

(3) Huhmann, B. L.; Neumann, A.; Boyanov, M. I.; Kemner, K. M.; Scherer, M. M. Emerging investigator series: As(V) in magnetite: Incorporation and redistribution. *Environ. Sci. Process Impacts* **2017**, *19* (10), 1208-1219.

(4) Perez, J. P. H.; Tobler, D. J.; Freeman, H. M.; Brown, A. P.; Hondow, N. S.; van Genuchten, C. M.; Benning, L. G. Arsenic species delay structural ordering during green rust sulfate crystallization from ferrihydrite. *Environ. Sci.: Nano* **2021**, *8*, 2950-2963.

(5) Perez, J. P. H.; Tobler, D. J.; Benning, L. G. Synergistic inhibition of green rust crystallization by co-existing arsenic and silica. *Environ. Sci.: Process. Impacts* **2024**, *26* (3), 632-643.

(6) Klementiev, K.; Chernikov, R. XAFSmass: A program for calculating the optimal mass of XAFS samples. *J. Phys. Conf. Ser.* **2016**, *712*, 012008.

(7) Diaz-Moreno, S.; Amboage, M.; Basham, M.; Boada, R.; Bricknell, N. E.; Cibin, G.; Cobb, T. M.; Filik, J.; Freeman, A.; Geraki, K.; Gianolio, D.; Hayama, S.; Ignatyev, K.; Keenan, L.; Mikulska, I.; Mosselmans, J. F. W.; Mudd, J. J.; Parry, S. A. The Spectroscopy Village at Diamond Light Source. *J. Synchr. Radiat.* **2018**, *25* (Pt 4), 998-1009.

(8) Ravel, B.; Newville, M. ATHENA, ARTEMIS, HEPHAESTUS: Data analysis for X-ray absorption spectroscopy using IFEFFIT. *J. Synchr. Radiat.* **2005**, *12* (Pt 4), 537-541.

(9) Webb, S. M. SIXpack: A graphical user interface for XAS analysis using IFEFFIT. *Phys. Scr.* **2005**, *T115* (T115), 1011-1014.

(10) Newville, M. IFEFFIT: Interactive XAFS analysis and FEFF fitting. *J. Synchr. Radiat.* **2001**, *8* (Pt 2), 322-324.

(11) Rehr, J. J.; Albers, R. C.; Zabinsky, S. I. High-order multiple-scattering calculations of X-ray-absorption fine structure. *Phys. Rev. Lett.* **1992**, *69* (23), 3397-3400.

(12) Kitahama, K.; Kiriyama, R.; Baba, Y. Refinement of the crystal structure of scorodite. *Acta Crystallogr. B.* **1975**, *31* (1), 322-324.

(13) van Genuchten, C. M.; Addy, S. E.; Pena, J.; Gadgil, A. J. Removing arsenic from synthetic groundwater with iron electrocoagulation: an Fe and As K-edge EXAFS study. *Environ. Sci. Technol.* **2012**, *46* (2), 986-994.

(14) Mikutta, C.; Frommer, J.; Voegelin, A.; Kaegi, R.; Kretzschmar, R. Effect of citrate on the local Fe coordination in ferrihydrite, arsenate binding, and ternary arsenate complex formation. *Geochim. Cosmochim. Acta* **2010**, *74* (19), 5574-5592.

(15) Perez, J. P. H.; Okhrymenko, M.; Blukis, R.; Roddatis, V.; Mayanna, S.; Mosselmans, J. F. W.; Benning, L. G. Vivianite-parasymplesite solid solution: A sink for arsenic in ferruginous environments? *Geochem. Persp. Lett.* **2023**, *26*, 50-56.

(16) Paktunc, D.; Dutrizac, J.; Gertsman, V. Synthesis and phase transformations involving scorodite, ferric arsenate and arsenical ferrihydrite: Implications for arsenic mobility. *Geochim. Cosmochim. Acta* **2008**, *72* (11), 2649-2672.

(17) Kelly, S. D.; Hesterberg, D.; Ravel, B. Analysis of soils and minerals using X-ray absorption spectroscopy. In *Methods of Soil Analysis Part 5—Mineralogical methods*, Ulery, A. L., Drees, L. R. Eds.; SSSA Book Series No. 5, Soil Science Society of America, 2008.

(18) Downward, L.; Booth, C. H.; Lukens, W. W.; Bridges, F. A variation of the F-test for determining statistical relevance of particular parameters in EXAFS fits. *AIP Conf. Proc.* **2007**, *882* (1), 129-131.

(19) Bots, P.; Shaw, S.; Law, G. T. W.; Marshall, T. A.; Mosselmans, J. F. W.; Morris, K. Controls on the fate and speciation of Np(V) during iron (oxyhydr)oxide crystallization. *Environ. Sci. Technol.* **2016**, *50* (7), 3382-3390.

(20) Marshall, T. A.; Morris, K.; Law, G. T. W.; Livens, F. R.; Mosselmans, J. F. W.; Bots, P.; Shaw, S. Incorporation of uranium into hematite during crystallization from ferrihydrite. *Environ. Sci. Technol.* **2014**, *48* (7), 3724-3731.

(21) Xiong, Y.; Guilbaud, R.; Peacock, C. L.; Krom, M. D.; Poulton, S. W. Phosphorus controls on the formation of vivianite versus green rust under anoxic conditions. *Geochim. Cosmochim. Acta* **2023**, *351*, 139-151.

(22) Hansen, H. C. B.; Poulsen, I. F. Interaction of synthetic sulphate "Green rust" with phosphate and the crystallization of vivianite. *Clays Clay. Miner.* **1999**, *47* (3), 312-318.

(23) Kukkadapu, R. K.; Zachara, J. M.; Fredrickson, J. K.; Kennedy, D. W. Biotransformation of two-line silica-ferrihydrite by a dissimilatory Fe(III)-reducing bacterium: Formation of carbonate green rust in the presence of phosphate. *Geochim. Cosmochim. Acta* **2004**, *68* (13), 2799-2814.

(24) van Genuchten, C. M.; Behrends, T.; Dideriksen, K. Emerging investigator series: Interdependency of green rust transformation and the partitioning and binding mode of arsenic. *Environ. Sci. Process Impacts* **2019**, *21* (9), 1459-1476.

(25) Wang, Y.; Morin, G.; Ona-Nguema, G.; Brown, G. E., Jr. Arsenic(III) and arsenic(V) speciation during transformation of lepidocrocite to magnetite. *Environ. Sci. Technol.* **2014**, *48* (24), 14282-14290.

(26) Muehe, E. M.; Morin, G.; Scheer, L.; Pape, P. L.; Esteve, I.; Daus, B.; Kappler, A. Arsenic(V) incorporation in vivianite during microbial reduction of arsenic(V)-bearing biogenic Fe(III) (oxyhydr)oxides. *Environ. Sci. Technol.* **2016**, *50* (5), 2281-2291.

(27) Ogorodova, L.; Vigasina, M.; Mel'chakova, L.; Rusakov, V.; Kosova, D.; Ksenofontov, D.; Bryzgalov, I. Enthalpy of formation of natural hydrous iron phosphate: Vivianite. *J. Chem. Thermodyn.* **2017**, *110*, 193-200.

(28) Ayala-Luis, K. B.; Koch, C. B.; Hansen, H. C. B. The standard Gibbs energy of formation of Fe(II)Fe(III) hydroxide sulfate green rust. *Clays Clay. Miner.* **2008**, *56* (6), 633-644.

(29) Robie, R. A.; Hemingway, B. S. *Thermodynamic properties of minerals and related substances at 298.15 K and 1 bar (10^5 pascals) pressure and at higher temperatures*; 1995. DOI: 10.3133/b2131.

(30) Majzlan, J.; Grevel, K.-D.; Navrotsky, A. Thermodynamics of Fe oxides: Part II. Enthalpies of formation and relative stability of goethite (α-FeOOH), lepidocrocite (γ-FeOOH), and maghemite (γ-Fe_2O_3). *Am. Mineral.* **2003**, *88* (5-6), 855-859.

(31) Majzlan, J.; Navrotsky, A.; Schwertmann, U. Thermodynamics of iron oxides: Part III. Enthalpies of formation and stability of ferrihydrite (~$Fe(OH)_3$), schwertmannite (~$FeO(OH)_{3/4}(SO_4)_{1/8}$), and ε-$Fe_2O_3$. *Geochim. Cosmochim. Acta* **2004**, *68* (5), 1049-1059.

(32) Nordstrom, D. K.; Majzlan, J.; Konigsberger, E. *Thermodynamic Properties for Arsenic Minerals and Aqueous Species*; 2014. DOI: 10.2138/rmg.2014.79.4.

(33) Rard, J. A.; Wolery, T. J. The standard chemical-thermodynamic properties of phosphorus and some of its key compounds and aqueous species: An evaluation of differences between the previous recommendations of NBS/NIST and CODATA. *J. Solution Chem.* **2007**, *36* (11), 1585-1599.

(34) Elzinga, E. J.; Sparks, D. L. Phosphate adsorption onto hematite: An in situ ATR-FTIR investigation of the effects of pH and loading level on the mode of phosphate surface complexation. *J. Colloid Interface Sci.* **2007**, *308* (1), 53-70.

(35) Arai, Y.; Sparks, D. L. ATR–FTIR spectroscopic investigation on phosphate adsorption mechanisms at the ferrihydrite–water interface. *J. Colloid Interface Sci.* **2001**, *241* (2), 317-326.

(36) Capitelli, F.; Chita, G.; Ghiara, M. R.; Rossi, M. Crystal-chemical investigation of $Fe_3(PO_4)_2{\cdot}8H_2O$ vivianite minerals. *Z. Kristallogr. Cryst. Mater.* **2012**, *227* (2), 92-101.

(37) Frost, R. L.; Martens, W.; Williams, P. A.; Kloprogge, J. T. Raman and infrared spectroscopic study of the vivianite-group phosphates vivianite, baricite and bobierrite. *Mineral. Mag.* **2002**, *66* (6), 1063-1073.

(38) Martens, W. N.; Kloprogge, J. T.; Frost, R. L.; Rintoul, L. Site occupancy of Co and Ni in erythrite-annabergite solid solutions deduced by vibrational spectroscopy. *Can. Mineral.* **2005**, *43* (3), 1065-1075.

(39) Peulon, S.; Legrand, L.; Antony, H.; Chausse, A. Electrochemical deposition of thin films of green rusts 1 and 2 on inert gold substrate. *Electrochem. Commun.* **2003**, *5* (3), 208-213.

(40) Frost, R. L.; Martens, W.; Williams, P. A.; Kloprogge, J. T. Raman spectroscopic study of the vivianite arsenate minerals. *J. Raman Spectrosc.* **2003**, *34* (10), 751-759.

(41) Myneni, S. C. B.; Traina, S. J.; Waychunas, G. A.; Logan, T. J. Experimental and theoretical vibrational spectroscopic evaluation of arsenate coordination in aqueous solutions, solids, and at mineral-water interfaces. *Geochim. Cosmochim. Acta* **1998**, *62* (19), 3285-3300.

(42) Makreski, P.; Stefov, S.; Pejov, L.; Jovanovski, G. Theoretical and experimental study of the vibrational spectra of (para)symplesite and hornesite. *Spectrochim. Acta A* **2015**, *144*, 155-162.

(43) Müller, K.; Ciminelli, V. S. T.; Dantas, M. S. S.; Willscher, S. A comparative study of As(III) and As(V) in aqueous solutions and adsorbed on iron oxy-hydroxides by Raman spectroscopy. *Water Res.* **2010**, *44* (19), 5660-5672.

(44) Zheng, Q.; Tu, S.; Chen, Y.; Zhang, H.; Hartley, W.; Ye, B.; Ren, L.; Xiong, J.; Tan, W.; Kappler, A.; Hou, J. Micropore sites in ferrihydrite are responsible for its higher affinity towards As(III) relative to As(V). *Geochim. Cosmochim. Acta* **2023**, *348*, 27-40.

(45) Goldberg, S.; Johnston, C. T. Mechanisms of arsenic adsorption on amorphous oxides evaluated using macroscopic measurements, vibrational spectroscopy, and surface complexation modeling. *J. Colloid Interface Sci.* **2001**, *234* (1), 204-216.

(46) Voegelin, A.; Hug, S. J. Catalyzed oxidation of arsenic(III) by hydrogen peroxide on the surface of ferrihydrite: An in situ ATR-FTIR study. *Environ. Sci. Technol.* **2003**, *37* (5), 972-978.

(47) Bhandari, N.; Reeder, R. J.; Strongin, D. R. Photoinduced oxidation of arsenite to arsenate on ferrihydrite. *Environ. Sci. Technol.* **2011**, *45* (7), 2783-2789.

(48) Liu, J.; Cheng, H.; Frost, R. L.; Dong, F. The mineral tooeleite $Fe_6(AsO_3)_4SO_4(OH)_4 \cdot 4H_2O$ – An infrared and Raman spectroscopic study-environmental implications for arsenic remediation. *Spectrochim. Acta A* **2013**, *103*, 272-275.

(49) Jia, Y.; Xu, L.; Wang, X.; Demopoulos, G. P. Infrared spectroscopic and X-ray diffraction characterization of the nature of adsorbed arsenate on ferrihydrite. *Geochim. Cosmochim. Acta* **2007**, *71* (7), 1643-1654.

(50) Li, B.; Wei, D.; Zhou, Y.; Huang, Y.; Tie, B.; Lei, M. Mechanisms of arsenate and cadmium co-immobilized on ferrihydrite inferred from ternary surface configuration. *Chem. Eng. J.* **2021**, *424*, 130410.

(51) Gao, X.; Root, R. A.; Farrell, J.; Ela, W.; Chorover, J. Effect of silicic acid on arsenate and arsenite retention mechanisms on 6-L ferrihydrite: A spectroscopic and batch adsorption approach. *Appl. Geochem.* **2013**, *38*, 110-120.

(52) Zubair, Y. O.; Fuchida, S.; Tokoro, C. Insight into the mechanism of arsenic(III/V) uptake on mesoporous zerovalent iron–magnetite nanocomposites: Adsorption and microscopic studies. *ACS Appl. Mater. Interfaces* **2020**, *12* (44), 49755-49767.

(53) Gomez, M. A.; Assaaoudi, H.; Becze, L.; Cutler, J. N.; Demopoulos, G. P. Vibrational spectroscopy study of hydrothermally produced scorodite ($FeAsO_4 \cdot 2H_2O$), ferric arsenate sub-hydrate (FAsH; $FeAsO_4 \cdot 0.75H_2O$) and basic ferric arsenate sulfate (BFAS; $Fe[(AsO_4)_{1-x}(SO_4)_x(OH)_x] \cdot wH_2O$). *J. Raman Spectrosc.* **2010**, *41* (2), 212-221.
